# Nicotinamide riboside for peripheral artery disease: the NICE randomized clinical trial

**DOI:** 10.1038/s41467-024-49092-5

**Published:** 2024-06-13

**Authors:** Mary M. McDermott, Christopher R. Martens, Kathryn J. Domanchuk, Dongxue Zhang, Clara B. Peek, Michael H. Criqui, Luigi Ferrucci, Philip Greenland, Jack M. Guralnik, Karen J. Ho, Melina R. Kibbe, Kate Kosmac, Donald Lloyd-Jones, Charlotte A. Peterson, Robert Sufit, Lu Tian, Stephanie Wohlgemuth, Lihui Zhao, Pei Zhu, Christiaan Leeuwenburgh

**Affiliations:** 1 Northwestern University Feinberg School of Medicine, Department of Medicine, Chicago, IL USA; 2 Northwestern University Feinberg School of Medicine, Department of Preventive Medicine, Chicago, IL USA; 3 University of Delaware, Department of Kinesiology & Applied Physiology, Newark, DE USA; 4 Northwestern University Feinberg School of Medicine, Department of Biochemistry and Molecular Genetics, Chicago, IL USA; 5 University of California at San Diego, Division of Preventive Medicine, San Diego, CA USA; 6 National Institute on Aging, Division of Intramural Research, Baltimore, MD USA; 7 University of Maryland School of Medicine, Department of Epidemiology and Public Health, Baltimore, MD USA; 8 Northwestern University Feinberg School of Medicine, Department of Surgery, Chicago, IL USA; 9 University of Virginia, Department of Surgery, Charlottesville, VA USA; 10 Augusta University, Department of Physical Therapy, Augusta, GA USA; 11 University of Kentucky, Center for Muscle Biology, Lexington, KY USA; 12 Northwestern University Feinberg School of Medicine, Department of Neurology, Chicago, IL USA; 13 Stanford University, Department of Health Research and Policy, Palo Alto, CA USA; 14 University of Florida, Department of Physiology and Aging, Gainesville, FL USA

**Keywords:** Randomized controlled trials, Peripheral vascular disease

## Abstract

People with lower extremity peripheral artery disease (PAD) have increased oxidative stress, impaired mitochondrial activity, and poor walking performance. NAD+ reduces oxidative stress and is an essential cofactor for mitochondrial respiration. Oral nicotinamide riboside (NR) increases bioavailability of NAD+ in humans. Among 90 people with PAD, this randomized double-blind clinical trial assessed whether 6-months of NR, with and without resveratrol, improves 6-min walk distance, compared to placebo, at 6-month follow-up. At 6-month follow-up, compared to placebo, NR significantly improved 6-min walk (+7.0 vs. −10.6 meters, between group difference: +17.6 (90% CI: + 1.8,+∞). Among participants who took at least 75% of study pills, compared to placebo, NR improved 6-min walk by 31.0 meters and NR + resveratrol improved 6-min walk by 26.9 meters. In this work, NR meaningfully improved 6-min walk, and resveratrol did not add benefit to NR alone in PAD. A larger clinical trial to confirm these findings is needed. Clinical Trials.gov registration: NCT03743636.

## Abstract (web summary)

In peripheral artery disease (PAD), this randomized trial assessed whether nicotinamide riboside (NR), with and without resveratrol, improved walking, compared to placebo. Here, the authors show that NR meaningfully improves 6-min walk, and resveratrol did not add benefit to NR alone.

## Introduction

People with lower extremity peripheral artery disease (PAD) have severe walking disability, but few effective treatments exist^1^. In PAD, lower extremity ischemia causes insufficient oxygen and nutrient delivery to lower extremity skeletal muscle, which increases oxidative stress, damages skeletal muscle fibers, and impairs mitochondria function.^2–7^.

NAD+ is an essential coenzyme for mitochondrial respiration and a co-substrate for enzymes involved in metabolic regulation, cellular stress resistance, and DNA damage repair, such as poly (ADP-ribose) polymerase (PARP)^8,9^. In preclinical study, NAD+ activated endothelial nitric oxide synthase (eNOS) and reduced oxidative stress to increase nitric oxide abundance^10^. Greater NAD+ abundance increased sirtuin1 (SIRT1) expression and improved skeletal muscle health, mitochondrial activity, and nitric-oxide-mediated endothelial function^11–13^. Nicotinamide riboside (NR) is a precursor to NAD+ and is available as an over-the counter supplement. In 2022, sales of NR in the U.S. exceeded 60 million dollars. In mice, oral NR, a precursor to NAD+, increased skeletal muscle SIRT-1 and mitochondrial activity and improved limb strength and running endurance^13,14^. In humans, oral NR increased NAD+ abundance^15,16^.

Resveratrol, a naturally occurring polyphenol, may increase SIRT1 affinity for NAD+^17^. Therefore, the **NIC**otinamid**E** riboside with and without resveratrol to improve functioning in PAD (**NICE**) clinical trial tested the hypotheses that NR alone improves 6-min walk distance, compared to placebo, and that NR + resveratrol improves 6-min walk distance, compared to placebo, in people with PAD.

Here we show that NR meaningfully improves 6-min walk, and resveratrol does not add benefit to NR alone in PAD.

## Results

**Fig. 1:**
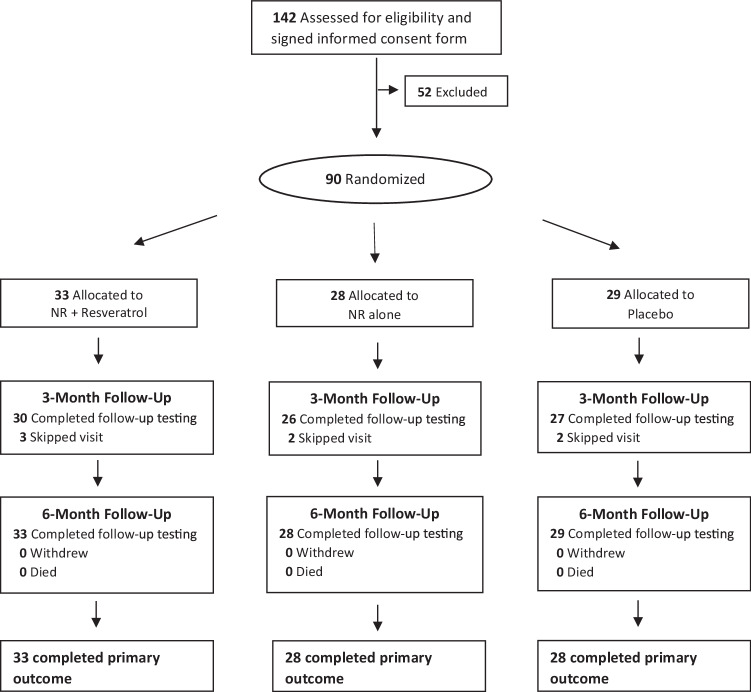
NICE Trial consort diagram. The figure shows the number of participants randomized to each study group and the number of people completing follow-up testing within each group.

Ninety participants were randomized and 89 (98.9%) completed 6-month follow-up (Fig. 1). Of 104 people who began run-in, eight (7.7%) did not meet criteria for passing and were excluded (Supplementary Table 1). Of 90 randomized, mean age was 71.2 years (standard deviation (SD): 9.1), 47.8% were Black, and 46.7% were female (Table 1). Proportions of participants with at least 75% adherence to pills were 75% for NR, 52% for NR + resveratrol, and 76% for placebo.

**Table 1:** Baseline characteristics of study participants with peripheral artery disease (*N* = 90)

Baseline variable	Trial assignment		
	NR+resveratrol	NR alone	Placebo
	(*N* = 33)	(*N* = 28)	*(N* = 29)
Age (years), mean (SD)	70.73 (8.97)	73.21 (9.97)	69.72 (8.29)
Male, *n* (%)	19 (57.58)	16 (57.14)	13 (44.83)
Female, *n* (%)	14 (42.42)	12 (42.86)	16 (55.17)
African American, *n* (%)	19 (57.58)	8 (28.57)	16 (55.17)
Ankle brachial index, mean (SD)	0.67 (0.20)	0.72 (0.18)	0.64 (0.18)
Body mass index (kg/m^2^), mean (SD)	29.30 (6.66)	28.65 (3.61)	30.37 (6.39)
Current smoker, *n* (%)	9 (27.27)	7 (25.00)	10 (34.48)
Former smoker, *n* (%)	19 (57.58)	16 (57.14)	13 (44.83)
Myocardial infarction, *n* (%)	6 (18.18)	3 (10.71)	5 (17.24)
Heart failure, *n* (%)	3 (9.09)	3 (10.71)	5 (17.24)
Stroke, *n* (%)	9 (27.27)	1 (3.57)	8 (27.59)
Angina, *n* (%)	5 (15.15)	3 (10.71)	2 (6.90)
Pulmonary disease, *n* (%)	8 (24.24)	8 (28.57)	9 (31.03)
Diabetes, *n* (%)	15 (45.45)	12 (42.86)	16 (55.17)
Intermittent claudication, *n* (%)	4 (12.12)	4 (14.29)	6 (20.69)
Atypical leg symptoms other than intermittent claudication, *n* (%)	24 (72.73)	20 (71.43)	17 (58.62)
No exertional leg pain, *n* (%)	5 (15.15)	4 (14.29)	6 (20.69)
6-min walk distance (meters), mean (SD)	336.61 (80.75)	339.18 (103.07)	325.60 (112.51)
Total treadmill walking time (min), mean (SD)^a^	9.94 (5.26)	7.29 (4.08)	7.11 (3.44)
WIQ distance score, mean (SD)	40.83 (31.66)	45.12 (28.20)	43.47 (24.85)
WIQ speed score, mean (SD)	41.40 (25.52)	47.01 (27.16)	43.40 (20.50)
WIQ stair-climbing score, mean (SD)	57.70 (31.32)	56.10 (27.18)	52.30 (30.55)

*SD* standard deviation.

^a^Sample sizes for treadmill walking time were 16 for NR alone, 17 for NR + resveratrol, and 14 for placebo. WIQ denotes walking impairment questionnaire and is scored on a 0–100 scale (100-best).

### Primary outcomes

**Fig. 2:**
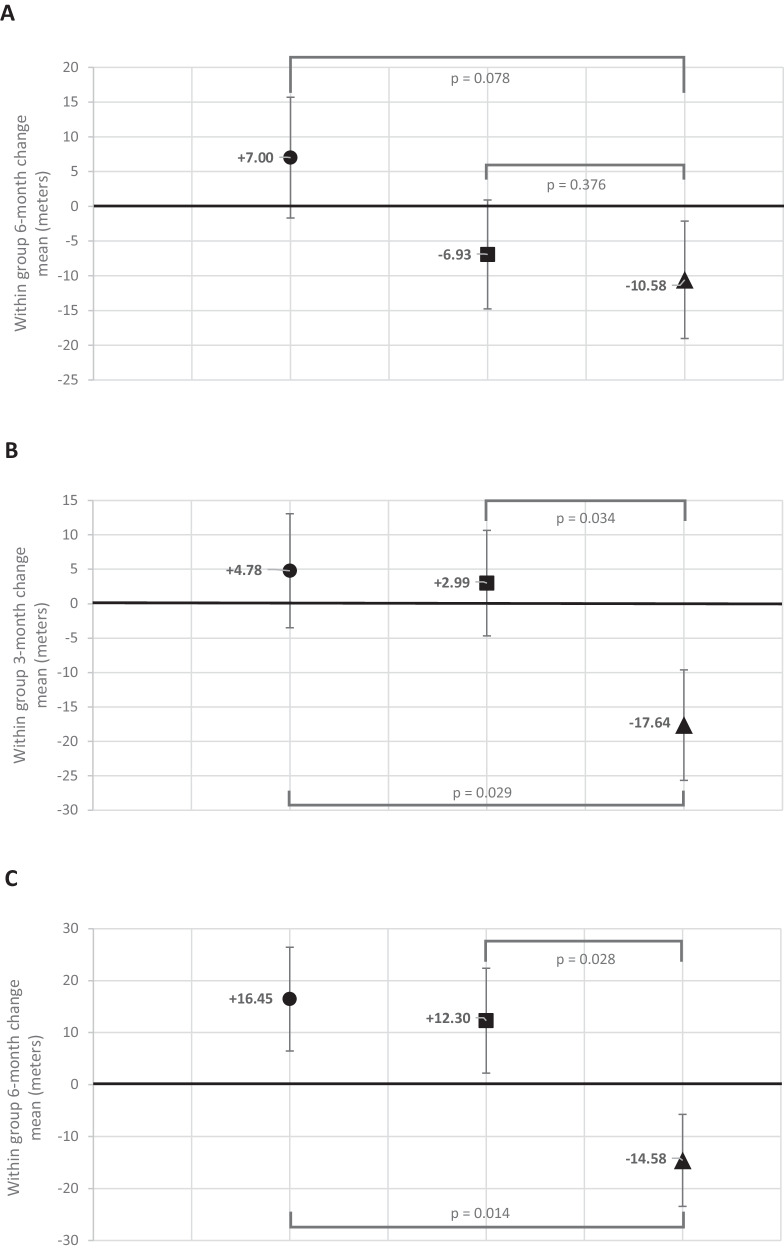
Mean change in 6-min walk distance by study group assignment. Figure **A** depicts the 6-month change in 6-min walk (primary outcome) results for *N* = 90 participants. Mixed models for repeated measures (MMRM) were used to compare 6-month change in 6-min walk distance between the NR + resveratrol and placebo groups and between the NR alone and placebo groups using baseline, 3-month, and 6-month values for 6-min walk distance, adjusting for age, sex, race, and baseline 6-min walk. The a priori statistical analysis plan defined a one-sided *P* value of <0.10 to define statistical significance. The circle represents NR alone, the square represents NR + resveratrol, and the triangle represents placebo. Figure **B** depicts 3-month change in 6-min walk (secondary outcome) for *N* = 90 participants. Mixed models for repeated measures (MMRM) were used to compare change in 6-min walk distance between the NR + resveratrol and placebo groups and between the NR alone and placebo groups using baseline, 3-month, and 6-month values for 6-min walk distance, adjusting for age, sex, race, and baseline 6-min walk. The a priori statistical analysis plan defined a one-sided *P* value of <0.10 to define statistical significance. The circle represents NR alone, the square represents NR + resveratrol, and the triangle represents placebo. Figure **C** depicts 6-month change in 6-min walk among the 60 participants who took at least 75% of study pills. Mixed models for repeated measures (MMRM) were used to compare 6-month change in 6-min walk distance between the NR + resveratrol and placebo groups and between the NR alone and placebo groups using baseline, 3-month, and 6-month values for 6-min walk distance, adjusting for age, sex, race, and baseline 6-min walk. The a priori statistical analysis plan defined a one-sided *P* value of <0.10 to define statistical significance. The circle represents NR alone, the square represents NR + resveratrol, and the triangle represents placebo.

Compared to placebo, NR improved 6-min walk by 17.6 meters (90% CI: + 1.77, +∞, *P* = 0.08) at 6-month follow-up, meeting the pre-specified criterion for statistical significance (Table 2 and Fig. 2A). Compared to placebo, NR+ resveratrol did not significantly improve 6-min walk at 6-month follow-up (+3.65 meters (90% CI:−11.2, +∞, *P* = 0.38)) (Table 2 and Fig. 2A).

**Table 2:** Effects of nicotinamide riboside alone and nicotinamide riboside with resveratrol on the primary outcome of 6-month change in 6-min walk in peripheral artery disease

	Nicotinamide riboside (NR)			Nicotinamide riboside (NR) + resveratrol (R)			Placebo				
	Baseline, mean (standard deviation)	6-mo. follow-up, mean (standard deviation)	Within group change, LSMeans (standard error)	Baseline, mean (standard deviation)	6-mo. follow-up, mean (standard deviation)	Within group change, LSMeans (standard error)	Baseline, mean (standard deviation)	6-mo. follow-up, mean (standard deviation)	Within group change, LSMeans (standard error)	NR vs. placebo, LSMeans (90% CI)	NR + R vs. placebo, LSMeans (90% CI)
6-min. walk distance (meters)	339.2 (103.1) *N* = 28	343.2 (100.0) *N* = 28	+7.0 (8.7)	336.6 (80.8) *N* = 33	328.9 (88.1) *N* = 33	−6.9 (7.8)	325.6 (112.5) *N* = 29	324.6 (89.6) *N* = 28	−10.6 (8.5)	+17.6 (+1.8, +∞) *P* = 0.08	+3.7 (−11.2, +∞) *P* = 0.38

Mixed models for repeated measures (MMRM) were used to compare 6-month change in 6-min walk distance between the NR + resveratrol and placebo groups and between the NR alone and placebo groups using baseline, 3-month, and 6-month values for 6-min walk distance, adjusting for age, sex, race, and baseline 6-min walk. One participant randomized to placebo had a very low baseline value but did not complete follow-up testing, contributing to the differences between the observed mean changes in 6-min walk distance from baseline to follow-up, compared to the least square mean estimate. The a priori statistical analysis plan defined a one-sided *P* value of <0.10 to define statistical significance.

### Secondary outcomes

At 3-month follow-up, compared to placebo, NR alone (+22.4 meters, (90% CI: +7.3,+∞), *P* = 0.029) and NR + resveratrol (+20.6 meters (90% CI: +6.3,+∞), *P* = 0.034) each significantly improved 6-min walk (Table 2, Fig. 2B). At 6-month follow-up, compared to placebo, NR significantly improved peak treadmill walking time (+2.1 min (90% CI: +0.24, +∞, *P* = 0.08)), while NR + resveratrol did not significantly improve treadmill walking time (+1.7 min (90% CI:−0.21, +∞, *P* = 0.12)) (Table 3). At 6-month follow-up, compared to placebo, NR alone did not significantly improve the WIQ distance score (−7.1 (90% CI:−15.2, +∞, *P* = 0.87)), physical activity total counts/day (+10,995 (90% CI:−2,078, +∞, *P* = 0.14)), or physical activity counts/min (+8.24 (90% CI:−23.85, +∞, *P* = 0.37)) (Table 3). At 6-month follow-up, compared to placebo, NR + resveratrol did not significantly improve the WIQ distance score (−5.1 (90% CI:−12.6, +∞, *P* = 0.80)), physical activity total counts/day (−842 (90% CI:−13,125, +∞, *P* = 0.54)), or physical activity counts/min (+7.47 (90% CI:−22.97, +∞, *P* = 0.38)) (Table 3).

**Table 3:** Effects of nicotinamide riboside alone and nicotinamide riboside with resveratrol on secondary outcomes of physical activity, the Walking Impairment Questionnaire and objectively measured walking performance in people with peripheral artery disease

	Nicotinamide riboside (NR)			Nicotinamide riboside (NR) + resveratrol (R)			Placebo				
	Baseline, mean (standard deviation)	Follow-up, mean (standard deviation)	Within group change, LSMeans (standard error)	Baseline, mean (standard deviation)	Follow-up, mean (standard deviation)	Within group change, LSMeans (standard error)	Baseline, mean (standard deviation)	Follow-up, mean (standard deviation)	Within group change, LSMeans (standard error)	NR vs. placebo, LSMeans (90% CI)	NR + R vs. placebo, LSMeans (90% CI)
WIQ distance score (0–100, 100-best)	45.1 (28.2) *N* = 28	42.9 (29.2) *N* = 28	−1.2 (4.4)	40.8 (31.7) *N* = 33	41.6 (30.8) *N* = 33	+0.82 (4.0)	42.2 (24.4) *N* = 28	48.6 (34.8) *N* = 28	+5.9 (4.3)	−7.1 (−15.2, +∞) *P* = 0.87	−5.1 (−12.6, +∞) *P* = 0.80
Physical activity – total activity counts per day	104,869 (61,260) *N* = 25	102,789 (58,695) *N* = 25	4,094 (7,109)	85,895 (33,530) *N* = 28	79,588 (40,235) *N* = 28	−7,743 (6,478)	86,396 (57,208) *N* = 25	83,567 (53,856) *N* = 25	−6,901 (6,889)	10,995 (−2,078, +∞) *P* = 0.14	−842 (−13,125, +∞) *P* = 0.54
Physical activity –counts per min	486.98 (166.55) *N* = 25	463.94 (140.56) *N* = 25	−8.81 (17.43)	458.38 (139.66) *N* = 28	448.73 (131.49) *N* = 28	−9.58 (16.00)	437.44 (116.09) *N* = 25	435.23 (124.84) *N* = 25	−17.05 (17.04)	8.24 (−23.85, +∞) *P* = 0.37	7.47 (−22.97, +∞) *P* = 0.38
6-min. walk distance at 3-month follow-up (meters)	339.2 (103.1) *N* = 28	330.9 (99.6) *N* = 24	+4.8 (8.3)	336.6 (80.8) *N* = 33	340.5 (82.4) *N* = 26	+3.0 (7.7)	325.6 (112.5) *N* = 29	324.0 (106.2) *N* = 24	−17.6 (8.0)	+22.4 (7.3, +∞) *P* = 0.029	+20.6 (6.3, +∞) *P* = 0.034
Max treadmill walking time at 6-month follow-up (min)^a^	7.2 (3.9) *N* = 10	8.7 (3.6) *N* = 10	+0.9 (0.9)	10.9 (5.0) *N* = 8	10.4 (4.2) *N* = 8	+0.6 (1.1)	7.1 (3.4) *N* = 8	6.1 (3.7) *N* = 8	−1.2 (1.0)	+2.1 (0.24, +∞) *P* = 0.08	+1.7 (−0.21, +∞) *P* = 0.12

For outcomes other than the 6-min walk test, analyses of covariance was used to compare differences between NR and placebo and between NR+ resveratrol and placebo, adjusting for age, sex, race, and the baseline value for each outcome. For the 6-min walk test, mixed models for repeated measures (MMRM) were used to compare change in 6-min walk distance between the NR + resveratrol and placebo groups and between the NR alone and placebo groups, adjusting for age, sex, race, and baseline 6-min walk. For the 6-min walk, one participant randomized to placebo had a very low baseline value but did not complete follow-up testing, contributing to the differences between the observed mean changes in 6-min walk distance from baseline to follow-up, compared to the least square mean estimate. The a priori statistical analysis plan defined a one-sided *P* value of <0.10 to define statistical significance.

^a^COVID-19 restrictions resulted in a lower sample size for the treadmill testing outcome.

Compared to placebo, the combined NR groups significantly improved 6-min walk distance at 3-month follow-up (+25.25 meters (90% CI: +10.43, +∞, *P* = 0.015)), maximal treadmill walking time at 6-month follow-up (+2.06 min (90% CI: + 0.24, +∞, *P* = 0.075)) and gastrocnemius muscle satellite cell abundance at 6-month follow-up (+11.14 (90% CI: +2.16, +∞, *P* = 0.060)) (Tables 4 and 5). Compared to placebo, the combined NR groups did not significantly improve 6-min walk distance (+14.05 meters, 90% CI: −1.25, +∞, *P* = 0.12), the WIQ distance score (−7.10 (90% CI:−15.19, +∞, *P* = 0.87), physical activity total activity counts/day (+10,995 (90% CI:−2,078, +∞, *P* = 0.14)), physical activity counts/min (+8.24 (90% CI:−23.85, +∞, *P* = 0.37)) or gastrocnemius muscle measures of NAD+ abundance or Percent Type I myofibers at 6-month follow-up (Tables 4 and 5).

**Table 4:** Effects of the combined nicotinamide riboside and nicotinamide riboside + resveratrol groups, compared to placebo, in participants with peripheral artery disease

	NR alone and NR + resveratrol groups combined			Placebo			Comparison of change between the combined NR groups and placebo
	Baseline, mean (standard deviation)	Follow-up, mean (standard deviation)	Within group change, LSMeans (standard error)	Baseline, mean (SD)	Follow-up, mean (standard deviation)	Within group change, LSMeans (standard error)	LSMeans (90% CI) *P* value
6-month change in 6-min walk distance (meters)	337.79 (90.88) *N* = 61	335.47 (93.23) *N* = 61	−0.24 (5.81)	325.60 (112.51) *N* = 29	324.58 (89.63) *N* = 28	−14.29 (9.97)	+14.05 (−1.25, +∞) *P* = 0.12
3-month change in 6-min walk distance (meters)	337.79 (90.88) *N* = 61	335.90 (90.27) *N* = 50	3.92 (5.61)	325.60 (112.51) *N* = 29	324.03 (106.23) *N* = 24	−21.34 (9.66)	+25.25 (10.43, +∞) *P* = 0.015
Maximal treadmill walking time (min)	8.84 (4.64) *N* = 18	9.46 (3.82) *N* = 18	0.73 (0.64)	7.14 (3.44) *N* = 8	6.13 (3.69) *N* = 8	−1.33 (1.28)	+2.06 (0.24, +∞) *P* = 0.075
WIQ distance score (0-100 scale, 100-best)	42.80 (29.96) *N* = 61	42.20 (29.82) *N* = 61	−0.21 (2.94)	42.23 (24.36) *N* = 28	48.60 (34.81) *N* = 28	6.89 (5.29)	−7.10 (−15.19, +∞) *P* = 0.87
Physical activity-total activity counts/day	94,845 (49064) *N* = 53	90,532 (50,669) *N* = 53	−1824 (4763)	86,396 (57,208) *N* = 25	83,567 (53,856) *N* = 25	−12819 (8528)	+10,995 (−2078, +∞) *P* = 0.14
Physical activity –counts per min	471.87 (152.11) *N* = 53	455.90 (134.74) *N* = 53	−9.20 (11.77)	437.44 (116.09) *N* = 25	435.23 (124.84) *N* = 25	−17.44 (20.95)	+8.24 (−23.85, +∞) *P* = 0.37

For the 6-min walk outcomes, mixed models for repeated measures (MMRM) were used to compare change in 6-min walk distance between the combined NR and NR + resveratrol groups and the placebo group, adjusting for age, sex, race, resveratrol randomization, and baseline 6-min walk. For other outcomes, analysis of covariance (ANCOVA) was used, adjusting for baseline values for each outcome, age, sex, race, and resveratrol randomization. The a priori statistical analysis plan defined a one-sided *P* value of <0.10 to define statistical significance.

**Table 5:** Effects of the combined nicotinamide riboside and nicotinamide riboside + resveratrol groups, compared to placebo, on gastrocnemius muscle biopsy outcomes in participants with peripheral artery disease (secondary outcomes)

	NR alone and NR + resveratrol groups combined			Placebo			Comparison of change between the NR groups and placebo
	Baseline, mean (standard deviation)	Follow-up, mean (standard deviation)	Within group change, LSMeans (standard error)	Baseline, mean (SD)	Follow-up, mean (standard deviation)	Within group change, LSMeans (standard error)	LSMeans (90% CI) *P* value
NAD+ (pmol/mg)	100.31 (68.33) *N* = 12	115.84 (60.95) *N* = 12	8.70 (17.92)	92.33 (59.14) *N* = 5	105.71 (46.95) *N* = 5	43.83 (26.15)	−35.13 (−81.20, +∞) *P* = 0.84
Satellite cell (satellite cells/100 fibers)	24.72 (17.63) *N* = 11	23.50 (12.44) *N* = 11	−0.81 (3.32)	18.30 (11.21) *N* = 5	12.27 (5.66) *N* = 5	−11.95 (5.18)	+11.14 (2.16, +∞) *P* = 0.060
Type 1 myofibers (%)	45.90 (21.73) *N* = 11	44.26 (17.77) *N* = 11	−2.88 (3.58)	56.79 (14.04) *N* = 5	50.34 (16.50) *N* = 5	−8.09 (5.75)	+5.21 (−4.47, +∞) *P* = 0.24

Analyses were performed with analysis of covariance (ANCOVA), adjusting for baseline values, age, sex, race, and randomization to resveratrol.

Compared to NR alone, NR + resveratrol did not significantly improve 6-min walk at 3-month follow-up or 6-min walk, maximal treadmill walking time or the WIQ distance score at 6-month follow-up (Table 6).

**Table 6:** Effects of nicotinamide riboside + resveratrol, compared to nicotinamide riboside, on outcomes among people with peripheral artery disease

	NR + resveratrol			NR alone			Comparison of change between the NR + resveratrol vs. NR
	Baseline, mean (standard deviation)	Follow-up, mean (standard deviation)	Within group change, LSMeans (standard error)	Baseline, mean (standard deviation)	Follow-up, mean (standard deviation)	Within group change, LSMeans (standard error)	LSMeans (90% CI) *P* value
6-month change in 6-min walk distance (meters)	336.61 (80.75) *N* = 33	328.93 (88.14) *N* = 33	−6.93 (7.84)	339.18 (103.07) *N* = 28	343.19 (99.97) *N* = 28	7.00 (8.69)	−13.93 (−29.15, +∞) *P* = 0.88
3-month change in 6-min walk distance (meters)	336.61 (80.75) *N* = 33	340.50 (82.42) *N* = 26	2.99 (7.67)	339.18 (103.07) *N* = 28	330.92 (99.63) *N* = 24	4.78 (8.28)	−1.78 (−16.51, +∞) *P* = 0.56
Maximal treadmill walking time (min)	10.86 (4.95) *N* = 8	10.40 (4.17) *N* = 8	0.57 (1.11)	7.23 (3.87) *N* = 10	8.71 (3.55) *N* = 10	0.89 (0.94)	−0.32 (−2.46, +∞) *P* = 0.58
WIQ distance score	40.83 (31.66) *N* = 33	41.62 (30.79) *N* = 33	0.82 (3.96)	45.12 (28.20) *N* = 28	42.88 (29.20) *N* = 28	−1.24 (4.42)	2.06 (−5.69, +∞) *P* = 0.37
Physical activity-total activity counts/day	85895 (33530) *N* = 28	79588 (40235) *N* = 28	−7743 (6478)	104869 (61260) *N* = 25	102789 (58695) *N* = 25	4094 (7109)	−11837 (−24396, +∞) *P* = 0.89
Physical activity –counts per min	458.38 (139.66) *N* = 28	448.73 (131.49) *N* = 28	−9.58 (16.00)	486.98 (166.55) *N* = 25	463.94 (140.56) *N* = 25	−8.81 (17.43)	−0.77 (−31.54, +∞) *P* = 0.51

For the 6-min walk outcome, between group comparisons at follow up visits (month 3 and month 6 visits) were based on a single mixed model for repeated measures (MMRM) analysis adjusted for baseline 6-min walk, age, sex, and race. For outcomes other than the 6-min walk, analyses were performed with analysis of covariance (ANCOVA), adjusting for baseline values, age, sex, and race. Baseline data include all randomized participants and the 3 and 6-month follow-up columns include only people who completed follow-up measurements. For the 6-min walk, one participant randomized to placebo had a very low baseline value but did not complete follow-up testing, contributing to the differences between the observed mean changes in 6-min walk distance from baseline to follow-up, compared to the least square mean estimate.

### Exploratory outcomes

At 6-month follow-up, compared to placebo, NR alone significantly increased gastrocnemius muscle satellite cell abundance (+11.14 satellite cells/100 fibers, 90% CI:+2.16, +∞, *P* = 0.06). At 6-month follow-up, compared to placebo, there was no significant effect of NR alone or NR + resveratrol on WIQ speed score, WIQ stair-climbing score, SF-36 physical functioning score, or on gastrocnemius muscle biopsy measures of NAD+ abundance, or myofiber type (Table 7). At 6-month follow-up, compared to placebo, NR + resveratrol did not significantly increase gastrocnemius muscle satellite cell abundance (Table 7). At 6-month follow-up, compared to NR alone, NR + resveratrol did not significantly improve WIQ speed score, WIQ stair climbing score, or SF-36 physical functioning score (Supplementary Table 4). At 6-month follow-up, compared to placebo, the combined groups of NR alone and NR + resveratrol did not significantly improve the WIQ speed score, the WIQ stair climbing score, or the SF-36 physical functioning score (Supplementary Table 3).

**Table 7:** Effects of nicotinamide riboside alone and nicotinamide riboside + resveratrol on exploratory muscle biopsy outcomes in people with peripheral artery disease

	Nicotinamide riboside (NR)			Nicotinamide riboside (NR) + resveratrol (R)			Placebo				
	Baseline, mean (standard deviation)	Follow-up, mean (standard deviation)	Within group change, LSMeans (standard error)	Baseline, mean (standard deviation)	Follow-up, mean (standard deviation)	Within group change, LSMeans (standard error)	Baseline, mean (standard deviation)	Follow-up, mean (standard deviation)	Within group change, LSMeans (standard error)	NR vs. placebo, LSMeans (90% CI)	NR + R vs. placebo, LSMeans (90% CI)
*Exploratory outcomes*											
Satellite cells (satellite cells/100 fibers)	28.04 (20.66) *N* = 7	25.76 (11.28) *N* = 7	1.73 (4.52)	18.90 (10.56) *N* = 4	19.53 (15.11) *N* = 4	−3.36 (4.33)	18.30 (11.21) *N* = 5	12.27 (5.66) *N* = 5	−9.41 (3.80)	11.14 (2.16, +∞) *P* = 0.060	6.05 (−1.86, +∞) *P* = 0.16
Type 1 fibers (%)	37.94 (19.57) *N* = 7	39.80 (19.29) *N* = 7	0.17 (4.86)	59.82 (20.04) *N* = 4	52.05 (13.45) *N* = 4	−5.94 (4.88)	56.79 (14.04) *N* = 5	50.34 (16.50) *N* = 5	−5.04 (4.12)	5.21 (−4.47, +∞) *P* = 0.24	−0.90 (−9.52, +∞) *P* = 0.56
NAD+ (pmol/mg)	85.82 (59.93) *N* = 7	96.68 (47.85) *N* = 7	−13.99 (23.44)	120.61 (81.06) *N* = 5	142.65 (72.38) *N* = 5	31.39 (22.57)	92.33 (59.14) *N* = 5	105.71 (46.95) *N* = 5	21.14 (20.03)	−35.13 (−81.20, +∞) *P* = 0.84	10.25 (−31.79, +∞) *P* = 0.37

Statistical comparisons were conducted using analysis of covariance (ANCOVA), adjusting for baseline values for each outcome, age, sex, and race. The a priori statistical analysis plan defined a one-sided *P* value of <0.10 to define statistical significance.

### Post-hoc analyses

Among all participants with 75% or greater adherence to pills, at 6-month follow-up, compared to placebo, NR improved 6-min walk by 31.0 meters (90% CI: + 13.2, +∞, *P* = 0.014) and NR + resveratrol improved 6-min walk by 26.9 meters (90% CI: 9.1, +∞, *P* = 0.028) (Fig. 2C). Among participants randomized to NR alone and among participants randomized to NR + resveratrol, those with 75% or greater adherence to study pills improved 6-min walk at 6-month follow-up, while those with <75% adherence declined in 6-min walk (Table 8). Among participants randomized to placebo, mean 6-min walk declined at 6-month follow-up, regardless of adherence (Table 8).

**Table 8:** Association of study drug adherence with change in 6-min walk at 6-month follow-up among participants with peripheral artery disease (*N* = 90)

	NR (75% were high adherers)		NR + resveratrol (52% were high adherers)		Placebo (76% were high adherers)	
	Adhered to ≥75% of study drug (*N* = 21)	Discontinued or adhered to ≤75% of drug (*N* = 7)	Adhered to ≥75% of drug (*N* = 17)	Discontinued or adhered to ≤75% of drug (*N* = 16)	Adhered to ≥75% of drug (*N* = 22)	Discontinued or adhered to ≤75% (*N* = 7)
6-month change in 6-min walk (meters)	+16.5	−13.8	+12.3	−25.5	−14.6	−3.7

Data shown are descriptive and no statistical testing was performed.

### Adverse events

Participants randomized to NR + resveratrol reported higher rates of diarrhea during the study (54.6%), compared to those randomized to NR alone (39.3%) and those randomized to placebo (27.6%). Participants randomized to NR + resveratrol also reported higher rates of nausea or emesis (36.4%) compared to those randomized to NR alone (14.3%) and placebo (24.1%) (Supplementary Table 5). One participant randomized to placebo experienced chest pain during the 6-min walk at three-month follow-up, was hospitalized, and later underwent coronary artery bypass grafting. No other serious adverse events were related to study participation.

## Discussion

In this double-blind randomized clinical trial of 90 participants with PAD, compared to placebo, NR improved 6-min walk distance by 17.6 meters at 6-month follow-up, consistent with a clinically meaningful effect^18,19^. Compared to placebo, NR significantly improved 6-min walk by 22.4 meters and NR + resveratrol significantly improved 6-min walk by 20.6 meters at 3-month follow-up. In post-hoc analyses, compared to placebo, NR alone significantly improved 6-min walk by 31.0 meters and NR + resveratrol improved 6-min walk by 26.9 meters among people with at least 75% adherence to study pills. NR increased satellite cell abundance in gastrocnemius muscle, compared to placebo, meeting the pre-specified criterion for statistical significance, but did not affect muscle fiber type.

To our knowledge, no prior randomized clinical trials have demonstrated beneficial effects of NR on walking performance in any human population. A clinical trial of 40 sedentary obese men showed no effect of NR 1000 mgs twice daily on changes in skeletal muscle NAD+ metabolites, insulin sensitivity, body composition, or mitochondrial activity^20–24^. Among 30 people with heart failure and ejection fraction <40%, NR 2000 mgs daily meaningfully increased plasma whole blood NAD+, but had no effect on 6-min walk, compared to placebo^24^. In contrast to participants in these prior clinical trials, PAD is characterized by lower extremity ischemia and increased lower extremity skeletal muscle reactive oxygen species (ROS)^2–4^.

While the magnitude of 6-min walk improvement at 6-month follow-up was greater in the NR alone group compared to the NR + resveratrol group, the difference between these two groups was not statistically significant. Among people randomized to NR alone, 75% took 75% or more study pills, while among those randomized to NR + resveratrol, only 52% took 75% or more of study pills. The poorer adherence among those randomized to NR + resveratrol explained the smaller effect of NR + resveratrol on improved 6-min walk, compared to placebo. In analyses of participants with at least 75% adherence, NR alone and NR + resveratrol each had similarly large and clinically meaningful effects on 6-min walk. Reasons for the lower adherence rate in people randomized to NR + resveratrol are unknown, but people randomized to NR + resveratrol reported higher rates of diarrhea and higher rates of nausea or emesis, compared to those randomized to NR alone or placebo. It is possible that study intervention adherence would have been poorer and overall effect sizes from NR may have been lower if successful completion of the run-in had not been required for eligibility.

This trial has several limitations. First, the sample size was relatively small. Results require confirmation in a larger study. Second, just 17 participants had muscle biopsy at baseline and follow-up, limiting statistical power to detect NR effects on muscle. Third, due to the COVID-19 pandemic, just 26 participants had treadmill testing at baseline and follow-up. Fourth, results may not be generalizable to potential participants who would not have passed the study run-in.

Among people with PAD, NR meaningfully improved 6-min walk at 6-month follow-up, and resveratrol did not add benefit to NR alone. Among participants with at least 75% adherence, the magnitude of the effect of NR on 6-min walk was comparable to the effects of supervised exercise for PAD. Further study is needed to confirm these findings in a larger cohort of participants with PAD.

## Methods

The Institutional Review Board of Northwestern University approved the protocol. Participants gave written informed consent. The study was a parallel-design, double-blinded, randomized clinical trial conducted in Chicago at Northwestern University Feinberg School of Medicine. Enrollment occurred between 12/13/2018 and 9/14/2022. The final follow-up occurred on 4/3/2023 when the last participant completed follow-up testing. Eligible participants were randomized to one of three groups: nicotinamide riboside 1,000 mg daily, nicotinamide riboside 1000 mg + 125 mgs resveratrol daily, or placebo. Enrollment was completed when the target sample size was attained. The study protocol is available in the supplementary information file as Supplementary Note 1.

### Participant identification

Participants were recruited with advertisements on Chicago-area buses and trains, postcards mailed to people aged 50 and older, and by contacting people with PAD who previously participated in research with the principal investigator (MMM) and expressed interest in future research. Participants received $25.00 for completing 6-month follow-up testing.

### Inclusion criteria

The inclusion criterion was presence of PAD, defined by an ankle brachial index (ABI) ≤0.90 in either leg^25,26^ or by medical record documentation of PAD from a vascular laboratory test obtained at a medical center or angiographic evidence of PAD (defined as 70% or greater stenosis in a lower extremity artery). Vascular laboratory testing criteria acceptable for inclusion included a toe brachial index <0.70, an ABI value ≤0.90, 70% or greater stenosis in a lower extremity artery on Duplex testing, or a post-exercise ABI decline of at least 20%.

### Exclusion criteria

Exclusion criteria included above or below-knee amputation, presence of critical limb ischemia, inability to walk without a wheelchair, requiring a walker for ambulation, presence of an ulcer on the bottom of either foot, end stage kidney disease, significantly impaired liver function, and failure to pass run-in. Participants whose walking was limited by a symptom other than PAD were excluded. Potential participants who planned any major surgery or lower extremity revascularization during the next 6 months and those who completed any major surgery, coronary revascularization, or lower extremity revascularization during the previous three months were excluded. Additional exclusion criteria included supervised treadmill exercise participation during the previous 6-months, participation in a randomized clinical trial during the previous three months, unstable angina, inability to communicate in English, pregnancy or pre-menopausal state, major medical illness, life-expectancy <6 months, dementia or a mini-mental status examination score <23^27^, and visual impairment limiting walking ability. People taking 250 mgs or more of nicotinamide riboside per day, vitamin B3, niacin, a slow release form of niacin, or resveratrol within the past 6 months were excluded.

### Run-in

The run-in was intended to identify and exclude potential participants who were unlikely to adhere to study interventions. Potential participants were asked to take five placebo pills daily (two NR placebo pills twice daily and one resveratrol placebo daily) for 14 days. Potential participants who did not take at least 70% of study pills were excluded.

### Randomization

A SAS program randomized eligible participants to one of three groups with equal probability (NR 1000 mgs alone, NR 1000 mgs + 125 mgs resveratrol, or placebo) using a randomly permuted block method with block sizes of four and six.

### Interventions

Interventions were administered in a double-blinded fashion for 6 months. Participants were asked to take five pills daily: Two 250 mgs of NR (or placebo) pills twice daily and one 125 mg resveratrol (or placebo) once daily. Participants were asked to record pill consumption on a paper log and return dispensed bottles at 3-month and 6-month follow-up\. Adherence was assessed using the medication log and pill counts.

### Nicotinamide riboside

NR (250 mg pills) and matching placebo were manufactured by ChromaDex.

### Resveratrol

Resveratrol capsules (125 mgs of 98% pure trans-resveratrol) and matching placebo were manufactured by ReserveAge.

### Ankle brachial index measurement

A hand-held Doppler probe (Pocket Dop II; Nicolet Biomedical Inc, Golden, CO) was used to obtain systolic pressures twice in the right and left brachial, dorsalis pedis, and posterior tibial arteries using established methods^25,26^.

### Medical history

Medical history was obtained using questionnaires administered by a trained and certified health interviewer^28^. Participant’s sex was designated based on the health interviewer’s assessment. Race was obtained from participants using an open-ended question with fixed categories.

### Leg symptoms

Leg symptoms were characterized using the San Diego claudication questionnaire^29^. Classical intermittent claudication (IC) was defined as exertional calf pain that did not begin at rest, caused the participant to rest, and resolved within ten min of rest^29,30^. Most people with PAD do not have classic IC symptoms^30^. People with PAD who report no exertional leg symptoms (i.e. asymptomatic) and those with exertional leg symptoms other than classic IC have significantly greater functional impairment and faster functional decline than people without PAD^1,30^. To enhance generalizability of results, participants without classic IC symptoms were included in the trial^30^.

### Primary outcome

The primary outcome was 6-month change in 6-min walk distance. There were two primary comparisons: Change in 6-min walk at 6-month follow-up between participants randomized to NR vs. placebo and change in 6-min walk at 6-month follow-up between participants randomized to NR + resveratrol vs. placebo.

### Secondary outcomes

Secondary outcomes were 3-month change in 6-min walk and 6-month change in maximal treadmill walking time, the Walking Impairment Questionnaire (WIQ) distance score, and daily physical activity measured by ActiGraph. Comparisons of 6-month changes in gastrocnemius muscle biopsy measures of NAD+ abundance, muscle phenotype, and satellite cell abundance between participants randomized to either NR or NR + resveratrol (combined) and placebo were secondary outcomes.

### Exploratory outcomes

Exploratory outcomes were 6-month change in the WIQ speed score, the WIQ stair climbing score, and the Short-Form-36 Physical Functioning score. Comparisons of 6-month change in gastrocnemius muscle biopsy measures of NAD+ abundance, muscle phenotype, and satellite cell abundance between NR and placebo and between NR + resveratrol and placebo were exploratory outcomes.

### 6-min walk test

Following a standardized protocol^28,31^, participants walked up and down a 100-foot hallway for six min after instructions, delivered by script, to cover as much distance as possible. Distance completed after six min was recorded. In PAD, a small minimum clinically important difference (MCID) was defined as ~8 meters and a large MCID as 20 meters^18^.

### Treadmill walking performance

Maximal treadmill walking time was measured using the Gardner-Skinner protocol at baseline and 6-month follow-up^32^. Treadmill testing was not available during the COVID-19 pandemic even after in-person visits became possible for other outcomes. Large MCID values for maximal treadmill walking time range from 2.5 to 4.0 min^19^.

### Walking Impairment Questionnaire Scores

The WIQ is a PAD-specific measure of self-reported limitations in walking distance, speed, and stair climbing, scored on a 0–100 scale (100 = best)^33^. An MCID for the WIQ was defined as approximately five points^19^.

### Health-related quality of life

The Short-Form 36 Physical Functioning (SF-36 PF) score measured health-related quality of life (range 0–100, 100 = best). The MCID is 5–7 points^34,35^.

### Physical activity

Free-living physical activity, measured as total counts per day, was acquired over seven days with the ActiGraph accelerometer^36^.

### Gastrocnemius skeletal muscle biopsy

An open muscle biopsy was performed in the medial head of the gastrocnemius muscle after subcutaneous lidocaine administration at baseline and 6-month follow-up^5–7,28^. Approximately 250 mgs of muscle tissue was removed. Samples were snap frozen in liquid nitrogen and stored at −80 degrees Celsius until testing.

### Gastrocnemius muscle measures

For measurement of NAD+ abundance, nucleotides were measured by High Performance Liquid Chromatography (HPLC) with a Shimadzu LC-20A pump and UV-VIS detector using a Supelco LC-18-Tcolumn, normalized to muscle weight (15 cm × 4.6 cm)^37^. Satellite cell abundance and fiber type were determined on frozen muscle sections using antibodies against: Pax7 (Pax7 concentrate, DSHB; 1:100), type 1 myosin (BA.D5 concentrate, DSHB; 1:100), and laminin (Millipore-Sigma, L9393; 1:100) as previously described^5,6^ (Supplementary Fig. 1).

### Other measures

Height and weight were measured at baseline. Body mass index (BMI) was calculated as weight (kg)/[height (meters)]^2^.

### Adverse events

Information about adverse and serious adverse events was obtained monthly with questionnaires.

### Sample size calculations

Anticipating a 90% follow-up rate, 30 participants randomized per group provided 80% power to detect a difference of 0.58 standard deviation (SD) of change in 6-min walk distance at a one-sided significance level of 0.10 based on a two-sample t-test^38^. Ninety participants provided more than 80% power to detect a 30-meter difference between NR + resveratrol and placebo and between NR alone and placebo at follow-up^38^. There was similar power to detect a 30-meter difference between the NR+ resveratrol group and NR alone groups. The study had 80% power to detect a between group difference of 0.58 standard deviations for other outcomes. The study had power to detect a 1.74 min difference in maximal treadmill walking time and 14.1 points for the WIQ distance score^38^.

### Statistical analyses

The two primary outcomes were the differences in 6-month change in 6-min walk distance between the NR and placebo groups and between the NR + resveratrol and placebo groups. Secondary analyses included the difference in 3-month change in 6-min walk distance between the NR and placebo groups, between the NR + resveratrol and placebo groups, between the NR + resveratrol and NR groups, and between the two NR groups combined and placebo. Secondary analyses included the difference in 6-month change in 6-min walk between the NR + resveratrol and NR groups and between the two NR groups combined and placebo. Secondary analyses included differences in 6-month change in maximal treadmill walking time, the WIQ distance score, and physical activity between the NR and placebo groups, between the NR + resveratrol and placebo groups, between the NR + resveratrol and NR groups, and between the combined NR + resveratrol and NR groups and placebo. Secondary analyses included differences in 6-month change in gastrocnemius biopsy measures between the two NR groups combined and placebo. Exploratory analyses compared change in exploratory outcomes at 6-month follow-up between the groups defined for secondary analyses and 6-month changes in the gastrocnemius muscle measures between the NR + resveratrol group and placebo and between the NR and placebo groups. In post-hoc analyses, differences in 6-month changes in 6-min walk distance were compared between the NR and placebo groups and between the NR + resveratrol and placebo groups among all participants with at least 75% adherence to pills.

Pre-specified analyses were performed according to each participant’s assigned group, irrespective of pill adherence. Baseline characteristics were summarized as means and standard deviations for continuous variables and frequencies and percentages for categorical variables. For the primary outcomes, mixed models for repeated measures (MMRM) were used to compare 6-month change in 6-min walk distance between the NR + resveratrol and placebo groups and between the NR alone and placebo groups using baseline, 3-month, and 6-month values for 6-min walk distance, adjusting for age, sex, race, and baseline 6-min walk. The a priori statistical analysis plan defined a one-sided *P* value of <0.10 to define statistical significance. One-sided 90% confidence intervals (CI) were calculated for between group differences. Because the clinical trial was a Phase II trial, to help ensure that a true beneficial effect of the interventions was not dismissed due to lack of statistical significance, the pre-specified *P* value for statistical significance was more liberal than a *P* value of <0.05. MMRM analysis included participants missing either 3-month or 6-month follow-up visits under the missing at random assumption and results were used for baseline to 3-month follow-up and baseline to 6-month follow-up. Analyses were repeated among people with at least 75% adherent. 6-month change in 6-min walk were analyzed for each group according to whether participants had 75% or greater adherence or <75% adherence, using MMRM. For outcomes other than 6-min walk, one-sided ANCOVA tests compared changes in each outcome, adjusting for age, sex, race, and the baseline value for each outcome. Analyses that compared the combined NR groups to placebo also adjusted for resveratrol randomization. There were no adjustments for multiple comparisons. Analyses were performed using SAS version 9.4.

### Reporting summary

Further information on research design is available in the Nature Portfolio Reporting Summary linked to this article.

### Supplementary information

Supplementary Information

Peer Review File

Reporting Summary

### Source data

Source data file

## Data Availability

The de-identified participant baseline characteristics, treatment assignments and outcome data generated in this study have been deposited in the American Heart Association GitHub database. Data can be accessed at 10.5281/zenodo.11099249. Data may be used for education and research purposes. The study protocol is available in the supplementary information file as Supplementary Note 1. Source data are provided with this paper.

## References

[1] Polonsky, T. S. & McDermott, M. M. Lower extremity peripheral artery disease without chronic limb-threatening ischemia: a review. *JAMA* **325**, 2188–2198 (2021). 34061140 10.1001/jama.2021.2126

[2] Weiss, D. J. et al. Oxidative damage and myofiber degeneration in the gastrocnemius of patients with peripheral arterial disease. *J. Transl. Med.* **11**, 230–239 (2013). 24067235 10.1186/1479-5876-11-230 PMC3849592

[3] Gillani, S., Cao, J., Suzuki, T. & Hak, D. J. The effect of ischemia reperfusion injury on skeletal muscle. *Injury* **12**, 670–675 (2012). 10.1016/j.injury.2011.03.008 21481870

[4] McDermott, M. M. et al. Skeletal muscle pathology in peripheral artery disease: a brief review. *Arterioscler. Thromb. Vasc. Biol.* **40**, 2577–2585 (2020). 32938218 10.1161/ATVBAHA.120.313831 PMC9571495

[5] Kosmac, K. et al. Correlations of calf muscle macrophage content with muscle properties and walking performance in peripheral artery disease. *J. Am. Heart Assoc.* **9**, e015929 (2020). 32390569 10.1161/JAHA.118.015929 PMC7660852

[6] White, S. H. et al. Walking performance is positively correlated to calf muscle fiber size in peripheral artery disease subjects, but fibers show aberrant mitophagy: an observational study. *J. Transl. Med.* **14**, 284 (2016). 27687713 10.1186/s12967-016-1030-6 PMC5043620

[7] Ferrucci, L. et al. Transcriptomic and proteomic of gastrocnemius muscle in peripheral artery disease. *Cir. Res.* **132**, 1428–1443 (2023). 10.1161/CIRCRESAHA.122.322325 PMC10213145 37154037

[8] Zapata-Perez, R., Wanders, R. J. A. & van Karnebeek, C. D. M. NAD+ homeostasis in human health and disease. *EMBO Mol. Med.* **13**, e13943 (2021). 34041853 10.15252/emmm.202113943 PMC8261484

[9] Freeberg, K. et al. Dietary supplementation with NAD+-boosting compounds in humans: current knowledge and future directions. *J. Gerontol: Med. Sci.* **78**, 2435–2448 (2023). 10.1093/gerona/glad106 PMC10692436 37068054

[10] Chen, Z. et al. Shear stress, SIRT1, and vascular homeostasis. *Proc. Natl Acad. Sci. USA* **107**, 10268–10273 (2010). 20479254 10.1073/pnas.1003833107 PMC2890429

[11] Elhassan, Y. S. et al. Nicotinamide riboside augments the aged human skeletal muscle NAD+ metabolome and induces transcriptomic and anti-inflammatory signatures. *Cell Rep.* **28**, 1717–1728 (2019). 31412242 10.1016/j.celrep.2019.07.043 PMC6702140

[12] de Picciotto, N. E. et al. Nicotinamide mononucleotide supplementation reverses vascular dysfunction and oxidative stress with aging in mice. *Aging Cell* **15**, 522–530 (2016). 26970090 10.1111/acel.12461 PMC4854911

[13] Zhang, H. et al. NAD+ repletion improves mitochondrial and stem cell function and enhances life span in mice. *Science* **352**, 1436–1443 (2016). 27127236 10.1126/science.aaf2693

[14] Khan, N. A. et al. Effective treatment of mitochondrial myopathy by nicotinamide riboside, a vitamin B3. *EMBO Mol. Med.* **6**, 721–731 (2014). 24711540 10.1002/emmm.201403943 PMC4203351

[15] Martens, C. R. et al. Chronic nicotinamide riboside supplementation is well-tolerated and elevates NAD+ in healthy middle-aged and older adults. *Nat. Commun.* **9**, 1286 (2018). 29599478 10.1038/s41467-018-03421-7 PMC5876407

[16] Trammell, S. A. J. et al. Nicotinamide riboside is uniquely and orally bioavailable in mice and humans. *Nat. Commun.* **7**, 12948 (2016). 27721479 10.1038/ncomms12948 PMC5062546

[17] Howitz, K. et al. Small molecule activators of sirtuins extend *Saccharomyces cerevisiae* lifespan. *Nature* **425**, 191–196 (2003). 12939617 10.1038/nature01960

[18] McDermott, M. M. et al. Meaningful change in 6-min walk in people with peripheral artery disease. *J. Vasc. Surg.* **73**, 267–276 (2021). 32335305 10.1016/j.jvs.2020.03.052 PMC9439935

[19] Gardner, A. W., Montgomery, P. S. & Wang, M. Minimal clinically important differences in treadmill, 6-min walk, and patient-based outcomes following supervised and home-based exercise in peripheral artery disease. *Vasc. Med.* **23**, 349–357 (2018). 29671381 10.1177/1358863X18762599 PMC6062461

[20] Dollerup, O. L. et al. A randomized placebo-controlled clinical trial of nicotinamide riboside in obese men. *Am. J. Clin. Nutr.* **108**, 343–353 (2018). 29992272 10.1093/ajcn/nqy132

[21] Dollerup, O. L. et al. Effects of nicotinamide riboside on endocrine pancreatic function and incretin hormones in nondiabetic men with obesity. *J. Clin. Endocrinol. Metab.* **104**, 5703–5714 (2019). 31390002 10.1210/jc.2019-01081

[22] Dollerup, O. L. et al. Nicotinamide riboside does not alter mitochondrial respiration, content or morphology in skeletal muscle from obese and insulin-resistant men. *J. Physiol.* **598**, 731–754 (2020). 31710095 10.1113/JP278752

[23] Conze, D., Brennar, C. & Kruger, C. Safety and metabolism of long-term administration of NIAGEN in a randomized, double-blinded, placebo-controlled clinical trial of healthy overweight adults. *Sci. Rep.* **9**, 9772 (2019). 31278280 10.1038/s41598-019-46120-z PMC6611812

[24] Wang, D. D. et al. Safety and tolerability of nicotinamide riboside in heart failure with reduced ejection fracture. *JACC Clin. Transl. Sci.* **7**, 1183–1196 (2022). 10.1016/j.jacbts.2022.06.012 PMC9831861 36644285

[25] McDermott, M. M. et al. Lower ankle/brachial index, as calculated by averaging the dorsalis pedis and posterior tibial arterial pressures, and association with leg functioning in peripheral arterial disease. *J. Vasc. Surg.* **32**, 1164–1171 (2000). 11107089 10.1067/mva.2000.108640

[26] Aboyans, V. et al. Measurement and interpretation of the ankle-brachial index: a scientific statement from the American Heart Association. *Circulation* **126**, 2890–2909 (2012). 23159553 10.1161/CIR.0b013e318276fbcb

[27] Heun, R., Papassotiropoulos, A. & Jennssen, F. The validity of psychometric instruments for detection of dementia in the elderly general population. *Int. J. Geriatr. Psychiatry* **13**, 368–380 (1998). 9658272 10.1002/(SICI)1099-1166(199806)13:6<368::AID-GPS775>3.0.CO;2-9

[28] McDermott, M. M. et al. Effect of low-intensity vs high-intensity home-based walking exercise on walk distance in patients with peripheral artery disease: the LITE randomized clinical trial. *JAMA* **325**, 1266–1276 (2021). 33821898 10.1001/jama.2021.2536 PMC8025122

[29] Criqui, M. H. et al. The correlation between symptoms and non-invasive test results in patients referred for peripheral arterial disease testing. *Vasc. Med.* **1**, 65–71 (1996). 9546918 10.1177/1358863X9600100112

[30] McDermott, M. M. Lower extremity manifestations of peripheral artery disease: the pathophysiologic and functional implications of leg ischemia. *Circ. Res.* **116**, 1540–1550 (2015). 25908727 10.1161/CIRCRESAHA.114.303517 PMC4410164

[31] McDermott, M. M. et al. 6-min walk is a better outcome measure than treadmill walking tests in therapeutic trials of patients with peripheral artery disease. *Circulation* **130**, 61–68 (2014). 24982117 10.1161/CIRCULATIONAHA.114.007002 PMC4154227

[32] Gardner, A. W. et al. Progressive vs. single-stage treadmill tests for evaluation of claudication. *Med. Sci. Sports Exerc.* **23**, 402–408 (1991). 2056896 10.1249/00005768-199104000-00003

[33] Regensteiner, J. G. et al. Evaluation of walking impairment by questionnaire in patients with peripheral arterial disease. *J. Vasc. Med. Biol.* **2**, 142–152 (1990).

[34] Ware, J. E., Snow, K. K., Kosinski, M. A. & Gandek, B. G. *SF-36 Health Survey: Manual And Interpretation Guide* (The Health Institute, New England Medical Center, 1993).

[35] Ward, M. M., Guthrie, L. C. & Alba, M. I. Clinically important changes in Short Form 36 Health Survey Scales for use in rheumatoid arthritis clinical trials: the impact of low responsiveness. *Arthritis Care Res.* **66**, 1783–1789 (2014). 10.1002/acr.22392 PMC4245332 24980417

[36] Kelly, L. A. et al. Validity of actigraphs uniaxial and triaxial accelerometers for assessment of physical activity in adults in laboratory conditions. *BMC Med. Phys.* **13**, 5 (2013). 24279826 10.1186/1756-6649-13-5 PMC4175111

[37] Peek, C. B. et al. Circadian clock NAD+ cycle drives mitochondrial oxidative metabolism in mice. *Science* **342**, 6158 (2013). 10.1126/science.1243417 PMC3963134 24051248

[38] McDermott, M. M. et al. Home-based walking exercise intervention in peripheral artery disease: a randomized clinical trial. *JAMA* **310**, 57–65 (2013). 23821089 10.1001/jama.2013.7231 PMC6007977

