# Publisher Correction: Nicotinamide riboside for peripheral artery disease: the NICE randomized clinical trial

**DOI:** 10.1038/s41467-024-51289-7

**Published:** 2024-08-12

**Authors:** Mary M. McDermott, Christopher R. Martens, Kathryn J. Domanchuk, Dongxue Zhang, Clara B. Peek, Michael H. Criqui, Luigi Ferrucci, Philip Greenland, Jack M. Guralnik, Karen J. Ho, Melina R. Kibbe, Kate Kosmac, Donald Lloyd-Jones, Charlotte A. Peterson, Robert Sufit, Lu Tian, Stephanie Wohlgemuth, Lihui Zhao, Pei Zhu, Christiaan Leeuwenburgh

**Affiliations:** 1 Northwestern University Feinberg School of Medicine, Department of Medicine, Chicago, IL USA; 2 Northwestern University Feinberg School of Medicine, Department of Preventive Medicine, Chicago, IL USA; 3 University of Delaware, Department of Kinesiology & Applied Physiology, Newark, DE USA; 4 Northwestern University Feinberg School of Medicine, Department of Biochemistry and Molecular Genetics, Chicago, IL USA; 5 University of California at San Diego, Division of Preventive Medicine, San Diego, CA USA; 6 National Institute on Aging, Division of Intramural Research, Baltimore, MD USA; 7 University of Maryland School of Medicine, Department of Epidemiology and Public Health, Baltimore, MD USA; 8 Northwestern University Feinberg School of Medicine, Department of Surgery, Chicago, IL USA; 9 University of Virginia, Department of Surgery, Charlottesville, VA USA; 10 Augusta University, Department of Physical Therapy, Augusta, GA USA; 11 University of Kentucky, Center for Muscle Biology, Lexington, KY USA; 12 Northwestern University Feinberg School of Medicine, Department of Neurology, Chicago, IL USA; 13 Stanford University, Department of Health Research and Policy, Palo Alto, CA USA; 14 University of Florida, Department of Physiology and Aging, Gainesville, FL USA

**Keywords:** Randomized controlled trials, Peripheral vascular disease

Correction to: *Nature Communications* 10.1038/s41467-024-49092-5, published online 13 June 2024

The abstract for this article was inadvertently truncated after the text ‘…A larger clinical trial to confirm these findings is needed.’; the following text was missing ‘Clinical Trials.gov registration: NCT03743636’. The original article has been updated.

