# Supplementary material for: Nicotinamide riboside for peripheral artery disease: the NICE randomized clinical trial
Source: Nat Commun. 2024 Jun 13;15:5046. doi: 10.1038/s41467-024-49092-5 (PMC11176364; doi:10.1038/s41467-024-49092-5)
Supplement: Supplementary file 1 — Supplementary Information [file 41467_2024_49092_MOESM1_ESM.pdf]

SUPPLEMENTARY NOTE 1: Study Protocol

PROTOCOL TITLE: NICotinamidE Riboside With and Without Resveratrol to Improve Functioning in Peripheral Artery Disease: The **NICE** Trial

PRINCIPAL INVESTIGATOR:

Mary McDermott, MD
Division of General Internal Medicine and Geriatrics
Department Medicine
312-503-6419
mdm608@northwestern.edu

VERSION NUMBER: Version #15.

VERSION DATE: 3/6/2023

STUDY SUMMARY:

Investigational Agent(s) (Drugs or Devices)	Nicotinamide riboside (NR)<br>Resveratrol
IND #	139436
Sample Size	90
Funding Source	American Heart Association (AHA)
Indicate the type of consent to be obtained	☒Written<br>☐Verbal/Waiver of Documentation of Informed Consent<br>☐Waiver of HIPAA Authorization<br>☐Waiver/Alteration of Consent Process
Research Related Radiation Exposure	☐Yes<br>☒ No
DSMB / DMC / IDMC	☒Yes<br>☐No

OBJECTIVES:

Our work shows that people with lower extremity peripheral artery disease (PAD) have greater functional impairment, faster functional decline, and higher rates of mobility loss compared to people without PAD (1-6). PAD-related ischemia-reperfusion of calf skeletal muscle during walking activity increases calf muscle oxidative stress and is associated with calf muscle fiber damage (7-12). Preliminary evidence also suggests that PAD is associated with calf muscle mitochondrial dysfunction (10-14). Calf muscle abnormalities are associated with functional impairment and mobility loss in people with PAD (15-18). No medical therapies have been identified that target these calf skeletal muscle abnormalities in PAD. Few medical therapies improve functioning or prevent functional decline in people with PAD.

Nicotinamide riboside (NR), a B vitamin and precursor to nicotinamide adenine dinucleotide (NAD+), significantly increases bioavailability of NAD+ in animals and humans (19). NAD+ is essential for mitochondrial respiration. NAD+ is also an essential cofactor for sirtuin1 (SIRT1)

activity (19-21). SIRT1 stimulates mitochondrial activity, activates endothelial nitric oxide synthase (eNOS) and reduces oxidative stress (22-29).

Oral administration of NR in mice increases skeletal muscle abundance of NAD+, increases skeletal muscle SIRT-1 activity, increases mitochondrial activity, and improves limb grip strength and running endurance (19,31-35). NR also increases muscle stem cell abundance and improves muscle fiber quality in mice (31,32). Therefore, we hypothesize that in people with PAD, NR will increase calf muscle mitochondrial activity, promote calf muscle regeneration, improve calf muscle quality, increase nitric oxide, and reduce calf muscle oxidative stress, thereby achieving better walking performance in PAD.

Resveratrol is a natural polyphenol and a supplement found in grape skin with pharmacological properties that also enhance mitochondrial activity through SIRT1 (30,36-40). Resveratrol increases SIRT1 affinity for its substrate and for NAD+. NAD+ is an essential cofactor for SIRT1 activity. Resveratrol also protects tissue against ischemia reperfusion injury (41,42). Because resveratrol increases SIRT1 affinity for NAD+, while NR increases calf muscle NAD+ abundance, we hypothesize that the combination of resveratrol and NAD+ will have a particularly potent stimulatory effect on SIRT-1 activity. Stimulating SIRT1 activity will increase calf skeletal muscle mitochondrial biogenesis, reduce oxidative stress, increase eNOS, and increase mitochondrial activity in people with PAD. In a randomized trial of 66 participants with PAD, we recently reported that 125 mgs of resveratrol increased the six-minute walk by 16.9 meters at 6-month follow-up, compared to placebo (P=0.07) (44). However, NAD+ is required for SIRT1 activity and our preliminary data show that people with PAD have lower NAD+ abundance than those without PAD (see Section B4). For these reasons, we hypothesize that combining resveratrol (a SIRT1 activator) with NR (which increases NAD+, an essential SIRT1 substrate) will have a particularly potent effect on calf skeletal muscle mitochondrial activity and walking performance in people with PAD.

The NICE Trial is a double-blind proof-of-principle randomized clinical trial to test two hypotheses: a) among people with PAD, NR significantly improves walking performance more than placebo and that b) among people with PAD, NR combined with resveratrol significantly improves walking performance more than placebo. In an exploratory aim, we will determine whether NR combined with resveratrol increases the six-minute more than NR alone. If our findings support our hypotheses, results will be used to design a large, definitive randomized clinical trial.

We will randomize 90 participants with PAD to one of the following three groups: NR + resveratrol, NR + placebo, or placebo + placebo. From this point forward, “NR alone” refers to NR + placebo and “placebo” refers to the combination of placebo+ placebo. Our primary outcome is change in six-minute walk between baseline and 6-month follow-up. Secondary outcomes are change in maximal treadmill walking time, patient-reported walking ability (measured by the Walking Impairment Questionnaire (WIQ)), and quality of life (measured by the Short Form-36 Physical Functioning (SF-36 PF) score). Calf skeletal muscle collected in participants in the NICE Trial will be analyzed for stem cell (satellite cell) abundance and muscle fiber phenotype.

BACKGROUND:

Lower extremity peripheral artery disease (PAD) is common and is associated with greater functional impairment, faster functional decline, and higher rates of mobility loss compared to the absence of PAD. PAD affects 8 million people in the U.S. and more than 200 million worldwide (45,46). Our work has established that people with PAD have greater

functional impairment and more rapid functional decline than those without PAD (1-6). PAD related functional impairment is associated with increased rates of hospitalization, nursing home placement, and mobility loss (5,47-49). Chronic disability, such as that associated with PAD, now accounts for more than half of the U.S. health burden (50). Yet therapeutic advances have not kept pace with the growing burden of disability from chronic disease (50).

Few effective medical therapies are available to improve walking ability in people with PAD. Only two medications, cilostazol and pentoxifylline, are FDA approved for treating PAD-associated walking impairment. Recently published American Heart Association (AHA) clinical practice guidelines for PAD recommend against pentoxifylline due to lack of efficacy (51). Benefits from cilostazol are modest and many patients with PAD discontinue cilostazol due to side effects (51-55). Most PAD patients are not candidates for revascularization (51,56). Even among PAD patients who undergo revascularization, up to 25-30% of lower extremity angioplasty/stent procedures fail within one year (57). Supervised treadmill exercise improves walking ability in patients with PAD, and the Center for Medicare and Medicaid Services (CMS) recently approved medical insurance reimbursement for 12 weeks of supervised treadmill exercise for people with PAD (58). However, most people with PAD opt not to participate in supervised exercise because of inconvenience or lack of interest (59). Furthermore, people with PAD live with disability for many years, while CMS covers just 12 weeks of supervised exercise. Identifying effective and safe therapies to improve walking performance and prevent mobility loss in PAD is an AHA priority that is directly addressed by the NICE Trial.

PAD is associated with ischemia-reperfusion injury of calf skeletal muscle. Patients with PAD experience calf muscle ischemia during walking, when metabolic demands exceed oxygen supply. Patients with PAD experience calf muscle reperfusion during rest, when blood supply increases sufficiently to meet calf muscle oxygen requirements. This phenomenon of ischemia-reperfusion generates reactive oxygen species (ROS), such as superoxide anion and hydrogen peroxide (7-14). ROS damage muscle fibers, impair mitochondrial activity, and promote apoptosis (7-14). Consistent with these phenomena, several small studies have described pathologic abnormalities in calf muscle of people with PAD (7,10,11,13-14). First, electron microscopy shows pathologic changes in inter-myofibrillar mitochondria, nuclei, and sarcolemma in calf muscle from patients with PAD (12). Second, several studies reported a quantitative mitochondria dysfunction in calf muscle of patients with PAD, with reduced energy production (10,13,14). Third, oxidative stress is increased in calf muscle of patients with PAD, compared to those without PAD (7). Among 34 participants with PAD and 21 without PAD who underwent calf muscle biopsy, PAD participants had a greater abundance of protein carbonyl content and 4-hydroxy-2-nonenal (HNE) ($P<.001$) than those without PAD, indicating a greater abundance of oxidative stress in calf muscle of participants with PAD compared to those without PAD. In the participants with PAD, greater abundance of oxidative stress was associated with more myofibrillar damage (7). Together these data suggest significant calf muscle pathology in patients with PAD, resulting in poorer mitochondrial activity, damaged myofibers, and reduced abundance of skeletal muscle fibers. We hypothesize that therapies reversing this calf muscle pathology will improve walking performance in patients with PAD. Consistent with our hypothesis, two recent studies in a mouse model of hind-limb ischemia showed that therapies targeting skeletal muscle mitochondrial damage significantly increased mitochondrial activity and improved skeletal muscle contractility (60,61).

Rationale for studying nicotinamide riboside (NR) in PAD. NR is rapidly metabolized to nicotinamide adenine dinucleotide (NAD+) and increases NAD+ abundance in animal and human tissue (19,31-35). We hypothesize that increasing NAD+ abundance in calf muscle of patients with PAD will improve their walking performance for the following reasons. First, NAD+

is essential for mitochondrial respiration and oxidative phosphorylation. Second, NR-induced increases in NAD+ promotes skeletal muscle regeneration in senescent animals by increasing skeletal muscle stem cells, called satellite cells (32). Third, NAD+ is an essential and rate limiting co-factor for SIRT1 activity, a histone deacetylation protein that promotes cell survival and longevity (31-35). SIRT1 has multiple beneficial functions that are likely to directly benefit people with PAD (27-29,62,63). Examples of how increasing SIRT1 activity is likely to improve walking performance in PAD are as follows. First, SIRT1 promotes mitochondrial activity by increasing abundance of PGC-1α, the master regulator of mitochondrial biogenesis and activity in muscle cells (28-30). In addition to promoting mitochondrial biogenesis, PGC-1α upregulates genes important for mitochondrial activity and stimulates angiogenesis (24,25,64). Second, increasing SIRT1 activity activates endothelial nitric oxide synthase (eNOS) and promotes angiogenesis (62,63). Third, increasing SIRT1 reduces cellular inflammation and oxidative stress by reducing NFkB (27-28,30). For these reasons, we hypothesize that by increasing NAD+ abundance, NR will increase calf muscle mitochondrial activity, improve calf muscle perfusion, and promote calf muscle regeneration in patients with PAD. Evidence from pre-clinical models to support our hypotheses follow.

NR activates SIRT1. Canto and colleagues fed 16 mice for one week with chow that contained either placebo or NR (31). The mice who ate NR enriched chow had a significantly greater abundance of NAD+ in their limb skeletal muscle than mice who ate placebo containing chow. Canto and colleagues then fed 40 mice chow that contained either placebo or NR for 16 weeks (31). Half of the mice received a normal diet and half received a high fat diet. After 12 weeks, the 20 mice receiving a high fat diet enriched with NR had significantly greater oxygen consumption and significantly greater treadmill running distance than those receiving a high fat diet with placebo. After 16 weeks, the mice fed the high fat diet were sacrificed. Mice receiving NR had greater mitochondrial activity and more abundant mitochondrial cristae in their gastrocnemius muscle, compared to mice receiving placebo. Mice receiving NR also had greater SIRT1 activation and greater PGC-1α activation, compared to mice receiving placebo. Canto and colleagues also observed greater expression of nuclear genes encoding regulators of oxidative metabolism in mice fed NR, but this phenomenon was not observed in mice fed placebo. None of the favorable changes in SIRT1 activity and none of the increases in mitochondrial activity were observed in the brain, where NAD+ did not increase. In summary, in a murine model, oral NR increased skeletal muscle NAD+ abundance and this increased NAD+ abundance increased SIRT1 activity, mitochondrial biogenesis, mitochondrial activity, treadmill running distance and oxygen consumption, compared to placebo.

NR promotes muscle regeneration, increases muscle quality, and increases mitochondrial respiration in aged mice (32). Zhang and colleagues fed young and aged mice NR enriched chow or regular chow for six weeks and reported the following results. First, in both young and old mice, mice that received NR enriched chow had a greater abundance of NAD+ in skeletal muscle stem cells compared to mice that received regular chow. Second, maximal running time, maximal running distance, and grip strength were significantly higher in aged mice fed NR enriched chow for six months, compared to aged mice fed plain chow for six weeks. Third, aged mice fed NR enriched chow had a significantly greater abundance of skeletal muscle stem cells compared to aged mice fed regular chow. Fourth, the aged mice fed NR enriched chow had significantly greater expression of genes responsible for mitochondrial respiration compared to aged mice fed plain chow. In summary, in aged mice, NR increased skeletal muscle NAD+ abundance, increased genes responsible for mitochondrial respiration, increased abundance of muscle satellite cells, and increased maximal running time, running distance, and grip strength. These results support our hypotheses that NR will improve walking performance in PAD.

NR improves mitochondrial activity in animals with mitochondrial myopathy. Khan and colleagues fed mice with a mitochondrial myopathy either an NR enriched chow or a regular chow for 16 weeks (21). After 16 weeks, the mice fed NR had greater skeletal muscle mitochondrial biogenesis, ATP production capacity, and oxygen consumption, than mice fed the regular chow. On electron microscopy, the mice fed NR had a greater abundance of mitochondrial cristae, consistent with a greater volume of mitochondria, compared to the mice fed regular chow. These findings also support our hypotheses that NR will increase mitochondrial activity in people with PAD.

Evidence of NR-induced bioavailability of NAD+ in humans. Trammell and colleagues demonstrated meaningful increases in NAD+ in human tissue following oral NR administration (19). A single oral 1,000 mgs dose of NR (approximately 15 mg/kg) increased peripheral blood mononuclear cell concentrations of NAD+ by 2.7 fold in a 52 year old healthy male. After taking 1,000 mg of NR daily for seven days, NAD+ levels achieved sustained elevations of 2.0 to 15.0-fold in peripheral blood mononuclear cells compared to baseline. These elevations in NAD+ were measurable by 4.1 hours after ingesting the first dose of NR 1,000 mgs (19). Next, oral NR was administered to 12 healthy men and women for three consecutive days at three different doses (100 mgs, 300 mgs, and 1,000 mgs). Each dose was separated by seven days. Peripheral blood mononuclear cell levels of NAD+ increased in a dose-dependent fashion, with meaningful increases in NAD+ observed in peripheral blood mononuclear cells within 8 hours of NR administration. Elevated levels were sustained 23 hours later. There were no adverse events associated with the 1,000 mg dose (19). A separate randomized trial by consultant and collaborator Dr. Chris Martens demonstrated significantly improved peripheral vascular stiffness, measured by tonometry, in healthy participants without cardiovascular disease (65). In summary, orally administered NR significantly increases NAD+ bioavailability in humans.

In summary, preclinical evidence supports our hypotheses that orally administered NR will activate SIRT1 in skeletal muscle, increase expression of genes responsible for mitochondrial activity, and provide substrate for mitochondrial activity. Preclinical evidence also supports our hypotheses that NR administration will promote calf skeletal muscle regeneration and increase walking endurance in patients with PAD.

Rationale for studying NR combined with resveratrol. Resveratrol (3,5,4'-hydroxystilbene) is a natural polyphenol that is present in grape skin. Resveratrol changes the conformational shape of the SIRT1 protein, increasing affinity of SIRT1 for its substrate and for NAD+. NAD+ is an essential co-factor for SIRT1 activity. Resveratrol increases the affinity of SIRT1 for substrate by 35-fold and increases the affinity of SIRT1 for NAD+ by more than five-fold, lowering the Michaelis constant (Km) for both substrate and NAD+ (43). In animals, resveratrol improves skeletal muscle oxidative metabolism and increases mitochondrial biogenesis (30,36-40). For example, LaGouge and colleagues fed male mice resveratrol enriched chow or regular chow for 15 weeks. After 15 weeks, non-oxidative muscle fibers had larger and more abundant mitochondria with increased mitochondrial enzyme activity (30). After 15 weeks, mice fed a high fat diet enriched with resveratrol ran further on a treadmill than mice fed a high fat diet without resveratrol. Gene expression showed that gastrocnemius expression of PGC-1α and SIRT1 were higher in mice treated with resveratrol compared to control mice. In a randomized controlled cross-over trial of 11 obese men (mean age 52.5 ± 2.1), Timmers and colleagues demonstrated that 150 mgs of resveratrol therapy for 30 days significantly increased mitochondrial respiration, PGC-1α abundance ($P<0.05$), and SIRT1 abundance ($P<0.05$) (66).

We recently completed the RESTORE Trial, a randomized double-blinded pilot clinical trial in which 66 participants with PAD age 65 and older were randomized to 500 mgs of resveratrol, 125 mgs of resveratrol, or placebo (44). Growing evidence suggests that lower doses of resveratrol are more effective than higher doses (67-73). Specifically, at lower doses, resveratrol increases proteins associated with cell survival and reduces ischemic damage, while at higher doses resveratrol promotes apoptosis (68-73). Therefore, we tested a high (500 mgs) and a low (125 mgs) dose of resveratrol and we hypothesized that the lower dose (125 mgs) of resveratrol would be more effective than high dose. After six months, 125 mgs of resveratrol increased the six-minute walk distance (primary outcome) by 16.9 meters compared to placebo (P=0.07). However, 500 mgs of resveratrol did not change the six-minute walk compared to placebo (-1.6 meters, P=0.965). As noted above, NAD+ is an essential cofactor for SIRT1 activity. Our pilot data from 16 people with PAD and six without PAD showed lower NAD+ abundance in calf skeletal muscle of those with PAD (107.5 vs. 117.6 pmol/mg, P=0.24). Therefore, we expect that combining resveratrol with NR, which increases NAD+ abundance, will achieve greater improvement in the six-minute walk than we observed in the RESTORE Trial.

Knowledge gaps that the NICE Trial will fill. Despite extremely promising evidence in pre-clinical models, no studies have tested NR or NR combined with resveratrol in humans with cardiovascular disease. If NR alone and/or NR with resveratrol improves functional performance and prevents functional decline in PAD, these widely available, inexpensive, and well-tolerated therapies will have a major impact on preventing mobility loss and improving quality of life in the large and growing number of people with PAD. No prior studies have assessed the therapeutic efficacy of these supplements in people with PAD.

Innovation. First, no prior studies have tested whether NR alone or NR combined with resveratrol significantly improves walking performance in people with PAD or in any populations with cardiovascular disease. Second, no prior randomized trials in PAD have tested interventions that simultaneously increase SIRT1 activity and increase abundance of NAD+, an essential substrate for both SIRT1 and mitochondrial activity. Third, our plan to test the efficacy of NR while simultaneously measuring change in calf muscle biopsy abundance of NAD+ in response to the intervention is innovative.

STUDY ENDPOINTS:

Primary Aim #1. Among participants with PAD, we will determine whether NR alone improves six-minute walk performance at 6-month follow-up, compared to placebo. *We hypothesize that participants randomized to NR alone will have greater increases or less decline in six-minute walk at 6-month follow-up, compared to those randomized to placebo.*

Primary Aim #2. Among participants with PAD, we will determine whether NR combined with resveratrol improves six-minute walk performance at 6-month follow-up, compared to placebo. *We hypothesize that participants randomized to NR combined with resveratrol will have greater increases or less decline in six-minute walk performance at 6-month follow-up, compared to those randomized to placebo.*

Secondary Aim #1. Among participants with PAD, we will determine whether NR alone improves six-minute walk performance at 3-month follow-up, compared to placebo. *We hypothesize that participants randomized to NR alone will have greater increases or less decline in six-minute walk at 3-month follow-up, compared to those randomized to placebo.*

Secondary Aim #2. Among participants with PAD, we will determine whether NR combined with resveratrol improves six-minute walk performance at 3-month follow-up, compared to placebo. *We hypothesize that participants randomized to NR combined with resveratrol will have greater increases or less decline in six-minute walk performance at 3-month follow-up, compared to those randomized to placebo.*
Secondary Aim #3*.* Among participants with PAD, we will determine whether NR combined with resveratrol improves six-minute walk performance at 3-month follow-up and at 6-month follow-up, respectively, compared to NR alone. *We hypothesize that participants randomized to NR combined with resveratrol will improve six-minute walk at 3-month follow-up and at 6-month follow-up, compared to NR alone.*

Secondary Aim #4. Among participants with PAD, we will determine whether NR alone improves maximal treadmill walking time, the WIQ distance score, and physical activity at 6-month follow-up, compared to placebo. *We hypothesize that participants randomized to NR alone will have greater improvement or less decline in maximal treadmill walking time, the WIQ distance score, and physical activity at 6-month follow-up, compared to those randomized to placebo.*

Secondary Aim #5. Among participants with PAD, we will determine whether NR combined with resveratrol improves maximal treadmill walking time, the WIQ distance score, and physical activity at 6-month follow-up, compared to placebo. *We hypothesize that participants randomized to NR plus resveratrol will have greater improvement or less decline in maximal treadmill walking time, the WIQ distance score, and physical activity at 6-month follow-up, compared to those randomized to placebo.*

Secondary Aim #6. Among participants with PAD, we will determine whether those randomized to NR + resveratrol have greater improvement in each outcome defined in Secondary Aims #4 and #5, compared to those randomized to NR alone. *We hypothesize that participants randomized to NR combined with resveratrol will improve each outcome at 6-month follow-up, compared to NR alone.*

Secondary Aim #7. Participants with PAD who received NR alone and those who received NR + resveratrol will be combined into one group and compared to the group receiving placebo, to determine whether NR alone or NR + resveratrol (combined group) have greater improvement in six-minute walk distance at 3-month follow-up and at 6-month follow-up, compared to those randomized to placebo. *We hypothesize that the combined NR group will improve six-minute walk distance at 3-month follow-up and at 6-month follow-up, compared to placebo.*

Secondary Aim #8. Participants who received NR alone and those who received NR + resveratrol will be combined into one group and compared to the group receiving placebo, to determine whether those randomized to NR alone or NR + resveratrol (combined group) have greater improvement in maximal treadmill walking time, the WIQ distance score, physical activity, and calf muscle biopsy measured NAD+ abundance, satellite cell abundance, and muscle fiber phenotype, compared to the placebo group. *We hypothesize that the combined NR group will improve each outcome at six-month follow-up, compared to placebo.*

Exploratory Aim #1*.* We will perform calf muscle biopsies to determine whether among participants with PAD, those randomized to NR + resveratrol or NR alone have increased calf muscle NAD+ abundance, increased satellite cell abundance and improved muscle fiber phenotype, compared to the placebo group. We hypothesize that PAD participants randomized to either NR+ resveratrol or NR alone will achieve increases in each of these measures,

compared to those randomized to placebo. Additional calf muscle measures will be assessed in Specific Aim #1 of a separate project funded as part of this American Heart Association Strategically Funded Research Network (SFRN) (PI: C. Leeuwenburgh, PhD).

Exploratory Aim #2. Among participants with PAD, we will determine whether NR alone improves the WIQ speed and stair climbing scores and the Short-Form Physical Functioning score at 6-month follow-up, compared to placebo.

Exploratory Aim #3. Among participants with PAD, we will determine whether NR combined with resveratrol improves the WIQ speed and stair climbing scores and the Short-Form Physical Functioning score at 6-month follow-up, compared to placebo.

Exploratory Aim #4. Among participants with PAD, we will determine whether NR combined with resveratrol improves the WIQ speed and stair climbing scores and the Short-Form Physical Functioning score at 6-month follow-up, compared to NR alone.

Exploratory Aim #5. Participants who received NR alone and those who received NR + resveratrol will be combined into one group and compared to the group receiving placebo, to determine whether those randomized to NR alone or NR + resveratrol (combined group) have greater improvement in the WIQ speed and stair climbing scores and the Short-Form Physical Functioning score at 6-month follow-up, compared to placebo.

STUDY INVESTIGATIONAL AGENTS:
Participants will be randomized to one of three groups: NR + resveratrol, NR + placebo, or placebo + placebo. Chromadex will supply the NR and matching placebo to the Northwestern Medicine Investigational Pharmacy. Reserveage Organics will supply resveratrol and matching placebo to the Northwestern Medicine Investigational Pharmacy.

The pharmacist will dispense study drug according to group assignment. Study medication is dispensed in bottles labeled with the participant name and study identification number. Bottles will NOT be labeled with the group assignment, ensuring that both the research team collecting data and study participants are blinded to group assignment (i.e. double blinded status). Study pills are stored and handled according to the Northwestern Medicine Research Pharmacy SOP.

Dr. Mary McDermott is the holder of the IND for the study (IND#139436).

PROCEDURES INVOLVED:
Initial eligibility criteria will be assessed by telephone. Potential participants who remain eligible after the telephone screening will be scheduled for a baseline visit, where they will undergo additional testing to determine their eligibility for randomization.

Baseline testing. Participants will provide written informed consent at baseline. An ankle brachial index (ABI) will be performed. Questionnaires will be administered and physical functioning tests will be performed. A treadmill exercise stress test will be performed. A blood sample will be obtained and height and weight will be measured. If the participant agrees to the optional muscle biopsy, they will be asked to undergo a biopsy. Baseline testing will require testing at multiple visits performed over multiple days. Research procedures are described in more detail below.

Run-in. The run-in is designed to exclude people who may not be adherent to the study drugs. To help ensure that randomized participants are adherent to study drug, we will ask all potential participants to complete a two week run-in prior to randomization. For the run-in, participants will be asked to take five study pills (2 NR placebo pills twice daily and 1 resveratrol placebo pill daily) for two weeks. Potential participants who take less than 70% of their placebo pills (i.e. less than 10 of 14 resveratrol placebo pills and/or less than 40 NR placebo pills) will be excluded.

Randomization. We will randomize participants to NR + resveratrol, NR + placebo, or placebo + placebo using a SAS computer program. Participants will be randomized using randomly selected block sizes of 4 and 6, to ensure random allocation between the three groups. A data analyst will perform the randomization and the group assignment will be communicated directly to the Northwestern Medicine Investigational Pharmacy.

Intervention and placebo. Participants will be asked to take pills daily (NR/placebo and resveratrol/placebo according to randomization assignment). Participants randomized to either NR + placebo or NR + resveratrol will receive 1,000 mg of NR daily (250 mg each; total of 4 pills). Participants randomized to NR + resveratrol will receive 125 mg of resveratrol daily (1 pill daily). Bottles will be picked up by a study coordinator and given to the participant in-person or sent via mail.

Participants will be asked to keep a daily medication log to record each time they take their study pills. They will be asked to retain their pill bottles and provide the bottles to study staff so that remaining pills (if any) can be counted. Additionally, relatively inexpensive items that may help participants better adhere to study procedures may be purchased on a case-by-case basis to assist with maximizing adherence to study procedures. An example is a pill organizing box that helps participants to better adhere to study pills.

Participants will be given their study pills in three-month supplies.

In-Person Visits, Telephone Calls, and 3-month follow-up testing. Participants will be asked to come in to the medical center up to once per month to monitor adverse events and to assess adherence to study medication by reviewing the medication logs and pill count. If a participant does not come to the medical center, a telephone call will be made to monitor adverse events.

At three months, participants will be asked to return to the medical center to perform a six-minute walk, return their pill log and pill bottles, and to receive a new three-month supply of study pills. Participants may be asked to return for additional visits at the discretion of the investigator to monitor adherence or for safety during the study period.

Six-month follow-up testing. Participants will return after taking the study pills for six months to complete final follow-up testing. Measures and tests that were conducted at baseline will be repeated and the pill logs and bottles will be collected.

Some or all study measures may be repeated at baseline or follow-up for data quality (e.g. if a treadmill test must be stopped due to extremely high blood pressure before the patient completed the test is one potential example of why a measure may need to be repeated). In some cases, it may be necessary to take an additional, unscheduled blood pressure measurement. For instance, if a participant has high blood pressure during the ABI and investigators would like to double check their arm pressure measurement before performing the six-minute walk either at the same visit or at a subsequent visit. Determinations about blood

pressure checks will be made on a case-by-case basis in consultation with Dr. McDermott or Dr. Lloyd-Jones or other qualified personnel.

In some cases, participants may be asked to take study pills for longer than six months. For example, if a participant has an illness that prevents them from returning at six months for follow-up testing or if they are out of town during their six-month follow-up window. In these instances, participants will be asked to continue taking their study pills for more than six months. Participants may be asked to take up to an additional three months of study pills.

Research procedures:
Ankle Brachial Index (ABI). After the participant rests supine for five minutes, the right brachial, dorsalis pedis (DP), posterior tibial (PT) and left DP, PT, and brachial artery pressures are measured using a hand-held Doppler probe. Pressures are measured twice. The ABI is calculated for each leg by dividing the average of the DP and PT pressures by the average brachial pressure. If an ankle brachial index result yields an equivocal result or suggests that a participant's lower extremity arteries are calcified, investigator discretion may be used to order a lower extremity vascular test with toe pressures at Northwestern Memorial Hospital to determine eligibility at baseline. The test will repeat blood pressures in the arms and legs and will include a toe pressure measurement, which is performed by placing a small blood pressure cuff around the great toe and attaching a plethysmography probe (circulatory sensor) on the pulpy part of the tip of the great toe.

Questionnaire Administration. Participants will be administered IRB-approved study questionnaires by a trained and certified study coordinator. Health-related quality of life will be assessed with the SF-36 Physical Functioning questionnaire (SF-36) and the Walking Impairment Questionnaire (WIQ). Patient report will be used to document comorbidities and physical activity.

COVID-19 Questionnaire. Currently enrolled and past participants may be called to see if they are willing to complete a questionnaire related to the COVID-19 pandemic. The questionnaire will be completed over the telephone and will help investigators determine how the pandemic is affecting older adults with PAD and how physical activity levels are affected during this time.

Six-minute walk. In the six-minute walk, participants walk back and forth along a 100-ft hallway for six minutes after standardized instructions to complete as many laps as possible. Distance covered in six minutes is recorded.

Treadmill testing. The Gardner graded treadmill exercise test will be used to measure change in maximal treadmill walking time in response to the study intervention. Speed is maintained at 2.0 miles per hour (mph) and treadmill grade increases by 2.0% every two minutes. If patients cannot walk at 2.0 mph, a Modified Gardner will be used, where treadmill speed is started at 0.5 mph and increased by 0.5 mph every 2 minutes until the participant reaches 2.0 mph, after which the treadmill grade is increased every two minutes while the speed remains at 2.0 mph.

Measuring physical activity. Physical activity will be measured in all participants over 10 days at baseline and follow-up, using the ActiGraph accelerometer. Participants are asked to wear the ActiGraph on the right side of their beltline for 10 days, removing it only for bathing or sleeping. After 10 days, participants mail the accelerometer back in a stamped, pre-addressed envelope provided to them at their study visit. We also contact participants by telephone to ensure monitors are returned.

Blood collection and long-term storage. At baseline and six-month follow-up study visits, participants may have approximately 50 ml of blood drawn for processing and long-term storage at -70 degrees Celsius. Approximately 10% of participants selected by chance will have an additional set of 45 ml of blood drawn for quality assurance at baseline and six-month follow-up (total 95 ml at baseline, 90 ml at follow-up). Blood will be tested for kidney and liver function at baseline. Stored blood will await later analyses for biomarkers and other emerging blood markers related to peripheral artery disease that may change in response to the intervention. A subset of participants will be invited to have an extra 9 ml tube of blood obtained to measure senescent cells (i.e. P16+ cells). For participants who agree to the optional element, blood will be obtained in blood tubes supplied by Sapere Bio, labeled with study ID number, and sent to Sapere Bio (400 Park Offices Dr, Research Triangle Park, NC 27709) for measurement of senescent cells. Sapere Bio will either destroy or return to Northwestern the remaining blood after measuring senescent cells. Genetic testing may also be performed on stored DNA if the participant agrees to this optional study element on the consent document. Results of the genetic testing on the sample will be stored with other data collected. Samples will be labeled with the participant's study identification number and will not be stored with other health or identifying information. Information associated with the sample will be stored a secure database on password protected computers that are secured by Northwestern University firewalls. Access is limited to study staff. If the samples are shared with other researchers not part of the current study, the PI will grant permission to the other researchers to analyze the samples after receiving IRB approval. Samples will be identified with a study identification number and the other researchers will not have access to PHI. Results of testing on the blood samples for long-term storage will not be shared with the study participants. Results of kidney and liver functioning will be shared upon request or if a value is medically significant and requires follow-up.

Calf muscle biopsies. Muscle biopsies will be performed by co-investigator Robert Sufit, MD, a board-certified neurologist with > 30 years of experience performing muscle biopsies. Muscle biopsies are obtained in the medial head of the gastrocnemius muscle in the leg with lowest ABI, at the point that is 67% of the distance between the medial malleolus and the medial aspect of the proximal tibia. If for some reason the leg with the lowest ABI cannot be biopsied (for example, not enough muscle available or if the participant or study team prefers not to biopsy the leg with the lowest ABI), then the other leg may undergo biopsy. Anesthesia is achieved with subcutaneous lidocaine. Subcutaneous and adipose tissue are dissected. Approximately 250 mgs of muscle is removed and immediately prepared for long term storage at -70 degrees Celsius. Approximately 100 to 150 mgs of fat may be removed from the subcutaneous fat and approximately 100 to 150 mgs of fat may be removed from below the fascial line during the muscle biopsy. In patients with more fat tissue, up to approximately 250 mgs of fat will be removed from the subcutaneous fat and from below the fascial line, respectively, at the discretion of the physician performing the muscle biopsy. Muscle tissue is frozen directly in liquid nitrogen for protein analyses and also mounted in trigacanth gum on cork and snap frozen in liquid nitrogencooled isopentane for histochemical analysis. At six-month follow-up, we will repeat the biopsy adjacent to the original biopsy, identifiable by the scar.

All calf muscle measures will be compared between baseline and six-month follow-up. To measure *NAD+*, frozen muscle will be analyzed using High-Performance liquid chromatography (HPLC) (81). Satellite cell number will be measured in the muscle specimens before and after study interventions in Dr. Peterson's lab at the University of Kentucky. Immunohistochemical detection of Pax7, a satellite cell-specific transcription factor will be performed on 7 μm frozen muscle cross-sections. Pax7+ DAPI+ nuclei will be counted and expressed per fiber type (82).

Because NR reduces senescence markers in aged mouse muscle (32), the frequency of p16+ nuclei, an indicator of senescence, will also be determined, as well as co-localization of p16 and Pax7. Muscle fiber type and size will be quantified with myosin heavy chain-specific antibodies as described (75), to assess overall muscle fiber phenotype in response to the interventions. The frequency of regenerating muscle fibers will also be determined using an antibody against embryonic myosin heavy chain. Additional muscle measures not specified here may be performed in Dr. Peterson's and in Dr. Leeuwenburgh's laboratory, based on findings in other ongoing studies of calf skeletal muscle dysfunction in people with PAD. Measures of senescence, including P16+ cells, may be performed in fat tissue obtained during the muscle biopsy in the laboratory of Dr. Peterson and/or Dr. Tchkonia at Mayo clinic.

Other measures. Body mass index (BMI) will be assessed at baseline and follow-up by objectively measuring height and weight. A four-meter walk test will be administered at usual and fastest pace at the baseline and follow-up visits. Participants will be asked to perform the usual paced four-meter walk at usual pace and the "fast paced" four meter walk at their fastest pace. Each of these short walks will be performed twice. Participants will be asked to complete a series of standing balance tests and chair stands. Names and doses of participants' medications will be systematically recorded at baseline and at follow-up.

Microbiome. Study participants may be asked to provide a stool sample. Participants who agree to this optional element will be given supplies to swab the toilet paper after a bowel movement, place the swab into a tube labeled with their study ID number, and mail back the tube in a pre-stamped envelope within 48 hours of collection. Alternatively, the participant may be asked to bring the swab to a study visit to return in person, depending on their appointment schedule. This sample will be used to identify specific bacterial species whose relative abundance is either negatively or positively correlated with PAD and other patient characteristics, such as body mass index and PAD severity. Participants will be asked to complete a short diet and lifestyle questionnaire (self-administered or verbally completed in person or over the phone with a research study coordinator) during the week when the stool is collected. The stool sample will be mailed for analysis to the laboratory of Dr. Jack Gilbert, a Professor of Pediatrics and a Professor in the Center for Microbiome Innovation at the University of California San Diego. Some of the serum that is already collected as part of study blood collection procedures may be mailed to Dr. Gilbert's laboratory for analyses of microbial metabolites.

DATA AND SPECIMEN BANKING

Muscle specimens and blood specimens for long-term storage will be stored in a freezer belonging to Dr. McDermott's research program at Northwestern University, in the freezer farm in the basement of Olson Pavilion. Specimens will be stored for up to 70 years, after which they will be destroyed.

Specimens will be coded; meaning that a key will exist that can link the codes back to the direct subject identifiers. Each participant will be assigned a unique study ID number that can be traced back to the study participant. The muscle specimens and the blood samples that are stored will be labeled with this unique identifier and the date and time of the blood collection.

Only Dr. McDermott has control over release of study data or specimens. Any investigators seeking to analyze blood or muscle specimens must contact Dr. McDermott for permission. Each request, if it occurs, will be considered on a case-by-case basis. Dr. McDermott will obtain IRB approval prior to releasing any blood or muscle specimens for analysis, other than those tests specifically named in this application.

SHARING RESULTS WITH PARTICIPANTS

Participants will receive results of their ankle brachial index (ABI) test and will be provided with a "result letter" at the end of their baseline visit. ABI and stress test results will be mailed to the participant's physician upon request. They will not be provided with other study results routinely. However, participants will be notified of medically significant stress test, blood, or blood pressure results identified in the stress test or ABI.

STUDY TIMELINES

Each participant will participate in the study for approximately six months after randomization. We plan to enroll 90 participants over an approximately three-year period. Primary analyses are anticipated to be completed by approximately April 2022.

INCLUSION AND EXCLUSION CRITERIA

Inclusion criteria. All participants will have PAD. PAD will be defined as the following: 1) An ankle-brachial index (ABI) $\leq$0.90 at the baseline study visit or 2) Vascular laboratory evidence or angiographic evidence of PAD. Inclusion based on prior vascular laboratory evidence will be determined by the study principal investigator and includes, for example, a toe brachial index (TBI) $<$ 0.70, Duplex measure showing 70% stenosis or great, a post heel-rise or post-exercise ABI drop of 20% or greater, or ABI values $\leq$ 0.90. Angiographic evidence of PAD consists of a stenosis of 70% or greater in a lower extremity artery.

Exclusion criteria.

1. Above- or below-knee amputation.
2. Critical limb ischemia.
3. Wheelchair-bound or requiring a walker to ambulate.
4. Walking is limited by a symptom other than PAD.
5. Current foot ulcer on bottom of foot.
6. End stage renal disease defined as a GFR less than 20 ml/min/1.73 M^2.
7. Significant liver impairment defined as two or more hepatic function enzymes $>$3.0 times the upper limit of normal. [NOTE: participants who meet this criterion may undergo a re-test of hepatic function tests to determine whether initially elevated hepatic enzymes represented a transient or spurious phenomenon.]
8. Failure to successfully complete the 2-week study run-in.
9. Planned lower extremity revascularization, orthopedic surgery, or other major surgery during the next six months.
10. Lower extremity revascularization, orthopedic surgery, cardiovascular event, coronary revascularization, or other major surgery in the previous three months.
11. Participation in supervised treadmill exercise during the previous six months.
12. Major medical illness including renal disease requiring dialysis, lung disease requiring oxygen, Parkinson's disease, a life-threatening illness with life expectancy less than six months, or cancer requiring treatment in the previous two years. [NOTE: potential participants may still qualify if they have had treatment for an early stage cancer in the past two years and the prognosis is excellent. Participants who only use oxygen at night may still qualify.]
13. Mini-Mental Status Examination (MMSE) score $<$23 or dementia.
14. Participation in or completion of a clinical trial in the previous three months. [NOTE: after completing a stem cell or gene therapy intervention, participants will become eligible after the final study follow-up visit of the stem cell or gene therapy study so long as at least six months have passed since the final intervention administration. After completing a supplement or drug therapy (other than stem cell or gene therapy), participants will be eligible after the final study

follow-up visit as long as at least three months have passed since the final intervention of the trial.]
15. Currently taking a dose of 250 mg or more of nicotinomide riboside, vitamin B3, niacin. Currently taking a slow-release form of niacin. Currently taking resveratrol or has taken any these drug(s) in past six months. Participants taking a multi-vitamin will not be excluded if the dose of vitamin B3 is less than 250 mgs.
16. Increase in angina or angina at rest
17. Non-English speaking.
18. Visual impairment that limits walking ability.
19. Women who are pregnant or who are pre-menopausal will not be eligible.
20. In addition to the above criteria, investigator discretion will be used to determine if the trial is unsafe or not a good fit for the potential participant.

Potentially eligible participants will be advised that Medicare coverage for 12 weeks of supervised treadmill exercise is now available. Potential participants who have not participated in supervised treadmill exercise covered by Medicare and who desire to participate will be referred back to their physician for follow-up. These individuals may become eligible six months after they complete supervised treadmill exercise.

Vulnerable populations (fetuses, pregnant women, children, prisoners, and institutionalized persons) and adults unable to consent will not be included in the study.

PARTICIPANT POPULATION(S)

Accrual Number:	Category/Group: (Adults/Children Special/Vulnerable Populations)	Consented: Maximum Number to be Consented or Reviewed/Collected/Screened	Enrolled: Number to Complete the Study or Needed to Address the Research Question
Local	Adults 18 and older	1000	90
Study-wide	N/A		
Total:		1000	90

RECRUITMENT METHODS

Participants will be identified from among individuals with PAD who have participated previously in research conducted by Dr. McDermott and/or who have expressed an interest in participating in future studies conducted by Dr. McDermott. Participants who we screen for ongoing studies who may have PAD but are ineligible for that study and interested participating in a study may be screened for this study.

In addition, PAD participants may be identified from among consecutive patients diagnosed with PAD in the non-invasive vascular laboratory at Northwestern Medical Group (NMG). Dr. Mark Eskandari is medical director of the non-invasive vascular laboratory at NMG and will assist with identifying potential participants from the non-invasive vascular laboratory. As director of the vascular laboratory at NMG, Dr. Eskandari formally reads many of the non-invasive vascular laboratory tests. He maintains all non-invasive vascular test results in his vascular laboratory.

As director of the vascular laboratory, Dr. Eskandari could conceivably contact the patients whose test results are maintained in his laboratory. However, Dr. Eskandari prefers that the contact of potential participants in studies come from the physicians referring him for testing. Lists of patients who have undergone lower extremity arterial testing in the non-invasive vascular laboratory are generated monthly and e-mailed from NMG to Dr. McDermott. A research assistant will contact referring physicians of potential participants identified from the vascular laboratory via fax, phone, page, or electronic message (EPIC or e-mail), to ask for permission to contact their patient about the study. If a reply is not received within three weeks, up to five letters are mailed from Dr. McDermott about the research study. We have substantial experience with our recruitment methods for our previous or ongoing studies.

We will also obtain lists of consecutive patients with a diagnosis of lower extremity peripheral arterial disease and individuals at high risk for peripheral artery disease from Northwestern's Enterprise Data Warehouse (EDW). EDW lists will be provided by an individual who is employed by the Division of General Internal Medicine who has received training and permission to obtain the lists from the EDW.

Similar methods will be used as those described above, in which the patient's physician will be contacted via fax, telephone, page, or electronic message (EPIC or email) to ask for permission to contact their patient about the study. If a reply is not received within three weeks, up to five letters are mailed from Dr. McDermott about the research study. In the recruitment letters, recipients are asked to call us if they are interested in participation or if they do not want to be contacted further. Potential participants who do not call us within three weeks of the first mailed recruitment letter may be telephoned by study staff and invited to participate.

In addition, we may use newspaper, television, and radio advertising to identify potential participants for this study. We will also use brochures, flyers, or posters that we will post in relevant office practices and public areas. We may use advertising on public transportation (i.e. Chicago Transit Authority) or online advertisements.

We will obtain a list of patients who live in the Chicago area from a mass mailing company. Using this, we will send postcards to those individuals on the list. The postcards will instruct people to call a study number if they are interested.

Recruitment materials for methods referenced above will be submitted to the IRB for approval prior to their use.

We may also use CAPriCORN for recruitment. CAPriCORN is a PCORI-funded network of institutions in the Chicago area. The purpose of CAPriCORN is to assist investigators with recruitment for clinical trials. CAPriCORN has its own IRB (Chicago Area Institutional Review board - CHAIRb) and uses ICD-9 codes and the electronic health record to identify potential participants who have PAD. These patients with PAD will be mailed a recruitment letter that describes the trial and invites the patient to participate.

We may also obtain IRB approval at the University of Chicago and/or the Jesse Brown VA Medical Center to recruit patients from these medical centers. If IRB approval is obtained from U of C and JBVAMC, letters will be mailed by an approved staff member to potential patients with known PAD. Participants recruited through these methods at JBVAMC will sign a VA consent document and will undergo some study testing on-site at the VA. Study tests include questionnaires and functional performance measures.

COMPENSATION FOR PARTICIPATION IN RESEARCH ACTIVITIES
Participants will receive $25 for completing six-month follow-up testing.

If the participant undergoes the optional muscle biopsy portion of the study, they will receive $100 per muscle biopsy.

Participants will be given assistance and/or reimbursement for expenses related to travel such as parking, bus/train fare, taxi or rideshare (i.e. Uber/Lyft) fare, and mileage, if requested. A receipt will be required for reimbursement above $40. Participants will be provided up to $90 per visit for travel reimbursement. If they require the use of our taxi or rideshare service, we will estimate the fare on www.taxifarefinder.com or on the rideshare website. A one-way fare estimate must be less than or equal to $45 (i.e. round trip of $90) in order for the study to provide taxi service. In some instances, a participant's travel estimate may be within the $90 limit for their first visit, but may unexpectedly increase at a later visit due to price fluctuations with Uber/Lyft. In these instances, the study will continue to provide travel to participants and pay the increased travel fare. In addition, if after randomization, a participant becomes unable to attend study visits and requires transportation such as a shared ride service in order to continue participation, then the travel service will be provided, using investigator discretion, so that the randomized participant can continue in the trial. In these cases, the amount of travel using our taxi or rideshare service may exceed $90.

WITHDRAWAL OF PARTICIPANTS
We anticipate that participant withdrawal from the research without their consent will be infrequent. However, a potential example is if a participant develops symptoms during the study and the principal investigator feels that the symptoms could make the study unsafe for the participant to continue. In this circumstance, the participant would be advised to follow-up with his or her physician. If the participant refuses to follow-up with their physician, it may be necessary for the participant to be withdrawn without their consent.

Some participants may decide they no longer want to participate in the study intervention. These participants will still be asked to return for follow-up testing.

Participants may withdraw from the research at any time. If they decide to leave the research, they should contact the principal investigator, Dr. Mary McDermott. If they stop being in the research, already collected data may not be removed from the study database.

RISKS TO PARTICIPANTS
Risks associated with NR and resveratrol. NR has been studied in humans and no serious adverse events have been reported (19,65). Resveratrol has been tested in multiple studies of humans (44,66,76). A systematic review reported that resveratrol is generally safe with only gastrointestinal symptoms reported at high doses (i.e. > 1,000 mgs) (76). Participants will be administered questionnaires monthly to obtain data regarding any potential side effects. Safety will also be monitored by our DSMB.

Risks associated with the muscle biopsy. The muscle biopsy is associated with several potential risks. These include discomfort during the muscle biopsy procedure and for 1-2 days afterward, scarring from the muscle biopsy skin incision, bleeding (including a hematoma), and infection. Adverse effects of Lidocaine administered prior to the biopsy include pain at the injection site, allergic reaction (including swelling of the tongue or throat, wheezing, difficulty

breathing, or death), emotional excitement, and a temporarily lowered heart rate and blood pressure. However, these adverse effects of lidocaine are rare. In addition, potential participants who are asked to hold their anti-platelet therapy during the week leading up to the muscle biopsy procedure may experience a cardiovascular event related to the temporary discontinuation of the anti-platelet therapy. First, to minimize risk related to muscle biopsy, all participants undergoing muscle biopsy will receive a written handout regarding signs to watch for that may indicate wound infection. They will also be verbally instructed in this. Each participant will be instructed to call Dr. McDermott immediately if any signs of infection occur. Second, permission from the participant's physician will be required before participants are asked to discontinue anti-platelet therapy. Participants will be telephoned approximately seven days after the muscle biopsy. Participants who report any complaints about their biopsy site (such as significant pain or redness) will be scheduled for an evaluation of their biopsy site by a study physician.

Risks associated with the six-minute walk test, treadmill stress test, four-meter walks, balance, and chair stands. The physical functioning tests may be associated with muscle fatigue or soreness. These symptoms typically resolve with rest. These tests may be associated with the risk of falling, irregular heartbeat, heart attack, or coronary ischemia or dyspnea due to heart failure or lung disease. Rarely, falling may result in a fracture. However, the research assistant who will collect these data has been trained to prevent falling. The risk of a fracture secondary to a fall during the testing is less than 1 in 8,000. If a participant experiences chest pain, research assistants are trained to page Dr. McDermott immediately. If the chest discomfort does not immediately resolve with rest, participants are escorted to Northwestern's Emergency Department, which is located in the same building as the location of the tests. Dr. McDermott facilitates follow-up as appropriate, by contacting participants' physicians, for those who experience new chest discomfort during testing, for example. In our experience, the risk of chest discomfort is approximately 1 in 750. Symptoms or results from the treadmill stress test may lead to further testing or hospitalization or recommendations for procedures to improve blood flow to the heart.

Risks associated with ABI measurement. The ankle brachial index measurement consists of measuring systolic blood pressure in each extremity using a hand-held Doppler. The ABI is non-invasive, safe and does not have any known lasting side effects. During the ankle-brachial index test, participants may experience discomfort from the inflated blood pressure cuff. However, this discomfort resolves immediately when the cuff is released.

Risks associated with questionnaire administration. Participation includes a risk of loss of confidentiality regarding personal health information. However, all research staff have undergone formal human subjects training. They are trained to protect the privacy of research subject participants.

Risks associated with drawing blood. The potential risks of drawing blood include a bruise at the site of vein puncture, inflammation of the vein, and infection. Participants undergoing a blood draw may experience lightheadedness, dizziness, or fainting.

In addition to these risks, this research may cause harm in ways that are unknown. These may be a minor inconvenience or may be so severe as to cause death.

POTENTIAL BENEFITS TO PARTICIPANTS

Participants who are randomized to receive nicotinamide riboside could experience improved functional performance or less decline in functional performance, if our hypotheses are correct.

DATA MANAGEMENT AND CONFIDENTIALITY

Data is recorded using preprogrammed instruments and an electronic case report form using secure, HIPAA-compliant REDCap database software on servers maintained by Northwestern's Clinical and Translational Sciences Institute. We have substantial experience with REDCap.

Power and Sample Size Estimation. We will enroll 30 patients per group. Based on our experience with randomized trials in PAD, we anticipate that 27 participants in each group will complete 6-month follow-up and provide outcomes for the statistical analysis (44,74). For Primary Aim 1, we will use a two-sample t-test to compare the 6-month decline in six-minute walk distance between NR alone and placebo. Twenty-seven participants per group provides 80% power for detecting a difference of 0.69 standard deviation (SD) of the decline at the one-sided significance level of 0.1. Based on our GOALS Trial results (74), a 0.58 SD represents a difference of 30 meters in six-minute walk distance. Similarly, for Primary Aim 2, we will have 80% power for detecting a difference of 30 meters between NR combined with resveratrol vs. placebo. For Secondary Aim #1 and Secondary Aim #2, we will have 80% statistical power for detecting a difference of 30 meters between the NR alone vs. placebo group (Secondary Aim #1) and between the NR combined with resveratrol group vs. the placebo group (Secondary Aim #2) at 3-month follow-up. In our Secondary Aim #3, we will have 80% statistical power for detecting a difference of 30 meters between the NR combined with resveratrol group and the NR alone group at 3-month and at 6-month follow-up. Most prior studies have defined clinically meaningful changes in the six-minute walk as 20-50 meters (77-80). Thus, the study has sufficient statistical power for detecting a clinically meaningful difference under the same assumptions. The NICE Trial is a proof of principle study intended to collect evidence for a large randomized trial. For Secondary Aim #4, we will compare changes in maximal treadmill walking distance, the WIQ distance score and physical activity between NR alone vs. placebo. The study provides 80% power for detecting a difference of 0.58 SD. Similarly, in Secondary Aim #5, we will compare changes in maximal treadmill walking distance, the WIQ distance score and physical activity between NR + resveratrol and placebo. In our GOALS trial, 0.69 SD of 6-month decline in WIQ represented a difference of 14 points for the WIQ distance score (74). The difference in reported cut-off values in WIQ scores between high and low PAD performers is 76 minus 43, or 33 points (83). Therefore, the study should have sufficient statistical power for detecting clinically important differences in the WIQ distance score. For Secondary Aim 6, we will compare 6-month changes in maximal treadmill walking distance, the WIQ distance score, and physical activity between people randomized to NR combined with resveratrol, compared to NR alone. For Secondary Aim 7, we will compare 3-month and 6-month changes in six-minute walk distance between all people randomized to an NR group (i.e. those randomized to NR combined with resveratrol and those randomized to NR alone) and those randomized to placebo. For Secondary Aim 8, we will compare 6-month changes in maximal treadmill walking time, the WIQ distance score, and physical activity between all people randomized to an NR group (i.e. those randomized to NR combined with resveratrol and those randomized to NR alone) and those randomized to placebo. In Secondary Aims #7 and #8, we have 80% power for detecting a difference of 0.50 SD. The proposed mixed effects regression analysis has a higher power than the two-sample t-test and thus can detect even smaller differences.

Statistical Analyses. Analyses will be performed using the intention to treat principle. Prior to the analyses, the distributions of the variables (changes) will be examined. For our Primary Aim #1, we will use a mixed effects repeated measurements (MMRM) regression to compare changes in six-minute walk performance at 6-month follow-up between the NR alone vs. placebo groups, adjusting for age, sex, race, and baseline six-minute walk distance. Age, sex,

and race will be adjusted for because of their association with change in six-minute walk distance (see below). Similar analyses will be performed for Secondary Aims #1, #2, #3, and #7. For our Primary Aim #2, we will use a one-tailed t-test based on MMRM to compared changes in six-minute walk between NR combined with resveratrol vs. placebo. We will perform MMRM regression analysis with the change in six-minute walk distance at month 3 and month 6 as the correlated response variable with an unstructured covariance matrix to evaluate differences in change in six-minute walk distance between the two groups in each comparison, adjusting for potential baseline confounders specified as following: baseline six-minute walk performance, age, sex, and race. The mixed effect regression would include patients missing the 6 month follow-up visit, assuming missing at random. If either primary aim is positive, the clinical trial will be considered positive. A one-sided P value at P<0.10 will be considered statistically significant. We are selecting this P value because of the preliminary (pilot) nature of this work. For Secondary Aims #4, #5, #6, #8 and Exploratory Aims, we will use the analysis of covariance to compare the 6-month change of each outcome, separately. In each specific comparison, the baseline value for the outcome of interest (i.e. WIQ score, treadmill, physical activity) will be adjusted for (rather than the six-minute walk) in addition to age, sex and race. For the Secondary Aims #7 and #8, the NR + placebo groups will be combined with NR + resveratrol into one group and compared to the placebo group and the MMRM regression or analysis of covariance will additionally adjust for the resveratrol usage.
For all of the analyses, The MMRM regression includes treatment indicator, visit time (month 3 and month 6 as categorical factors), the interactions between treatment and visit time, baseline six minute walking distance (for all aims in which six-minute walk is the outcome), age, sex and race. For comparisons of other endpoints not measured longitudinally, the conventional analysis of covariance will be used adjusting for the baseline level of the corresponding outcome, age, sex, and race. We will adjust for baseline six-minute walk, age, sex, and race, because these variables have been correlated significantly with either change in six-minute walk or with six-minute walk at follow-up in our prior studies. For example, in the combined data from the control groups of our GOALS, LITE, PROPEL, and TELEX randomized clinical trials of exercise interventions for people with PAD, six-minute walk at baseline was correlated with six minute walk distance at six-month follow-up (Pearson correlation coefficient= 0.812, P<0.001). In addition, participants who were Black had significantly poorer six-minute walk, compared to those who were not Black, at six-month follow-up (320.9 meters (SD: 104.5) vs. 359.8 meters (102.5), P=0.0035). Women had significantly poorer six-minute walk, compared to men, at six-month follow-up (309.6 (SD: 103.2) vs. 358.7 (SD: 102.1), P<0.001). In our observational WALCS cohort of men and women with PAD, older age was associated significantly with greater decline in six-minute walk distance at one-year follow-up (correlation= -0.236, P<0.001).
In a sensitivity analysis, we will repeat all comparisons additionally adjusting for baseline covariates that show major imbalances between relevant treatment groups, where the major imbalance is defined as Cohen's effect size being greater than 0.5. The MMRM analysis for comparing the change in six-minute walking distance will be based on all participants who have their six minute walking distance measured at least once during follow-up (i.e. at least at month 3 or month 6).

Quality Control. Ten percent of participants are randomly identified for quality control. They will have their ABI measured twice by independent examiners. They will have a split sample of muscle tissue (i.e. two samples from one participant) sent for analyses. The second muscle sample is designated an arbitrary identification number to which the technician is blinded. Using these methods, quality control is monitored continuously.

PROVISIONS TO MONITOR THE DATA TO ENSURE THE SAFETY OF PARTICIPANTS

A Data and Safety Monitoring Board (DSMB) will monitor safety throughout the study. The DSMB consists of three internationally recognized scientists including: Dr. Thomas Pearson MD, FAHA Professor of Epidemiology, Dr. Joe Ix, Professor and Chief of the Division of Nephrology at University of California San Diego, and Dr. Michael Miller, Professor of Biostatistics at Wake Forest Baptist Medical Center. The DSMB will meet at least every six months during the study. The DSMB will review and approve the protocol prior to beginning data collection. They will decide on stopping criteria for the study. On July 13, 2108 during their initial meeting the DSMB decided that stopping criteria would be only for safety. The biostatisticians will work with the DSMB to perform interim analyses regarding safety only.

Hospitalizations will be reported to the DSMB within seven days of investigators learning of the event, if the hospitalizations are unexpected and may be related to study participation. All deaths will be reported to the DSMB within 24 hours. All new injuries or illness causing serious, chronic disability that are unexpected and that may be related to study participation will be reported to the DSMB within seven days of investigators learning of the event. At the DSMB meetings, held approximately every six months, a report of all hospitalizations by study group will be presented to the DSMB.

Laboratory testing will be performed at Northwestern Hospital and results will be e-mailed directly to Dr. McDermott through the Electronic Health Record. In addition, the laboratory pages Dr. McDermott with any serious alert values, according to the laboratory's criteria. Dr. McDermott will follow-up with study participants and arrange appropriate follow-up, depending on the alert value. If Dr. McDermott is not available, the physician covering for her will receive the telephone calls from the laboratory and arrange for appropriate follow-up.

PROVISIONS TO PROTECT THE PRIVACY INTERESTS OF PARTICIPANTS

Research staff undergo training (human subjects training) in the protection of participant confidentiality and privacy. Research staff have access to medical records only for the purpose of conducting research that is approved by the IRB. Research procedures will be conducted in an enclosed space by a trained and certified research assistant. Dr. McDermott certifies research assistants in data collection to help ensure that participants are treated with the highest level of professionalism.

COMPENSATION FOR RESEARCH-RELATED INJURY

If the participant needs medical care because of taking part in this research study, they should contact the investigator and medical care will be made available. This care will be billed to the participant, their insurance, or other third party. Northwestern University has no program to pay for medical care for research-related injury.

ECONOMIC BURDEN TO PARTICIPANTS

Describe any costs that participants may be responsible for because of participation in the research. All of the study procedures and measures are paid for by the investigative team.

CONSENT PROCESS

Written informed consent will be obtained from the participant prior to study procedures. Participants will be consented by a research assistant who has been trained and certified by Dr. McDermott in obtaining informed consent. A research assistant will explain the study to potential participants by telephone prior to their first study visit. When a potential participant arrives to the medical center for study participation, the research assistant will explain the full details of the research study. The informed consent process will take place at the initial baseline study visit in

a private area on Northwestern's medical campus. Potential participants will be provided plenty of time to read the consent form. The research assistant will answer questions and Dr. McDermott or another study investigator at Northwestern is also available to answer any questions that participants may have about the research. If the participant would like more time to discuss the research study with their physician or family member before signing the consent document, they will be allowed to do so and the study visit will be rescheduled for a later date. Potential participants who do not speak English, subjects who are not yet adults, cognitively impaired adults, and adults unable to provide written consent will not be eligible for study participation.

PROTECTED HEALTH INFORMATION (PHI AND HIPAA)

HIPAA Authorization will be obtained from all participants as part of the informed consent process.

QUALIFICATIONS TO CONDUCT RESEARCH AND RESOURCES AVAILABLE

The multidisciplinary investigative team for this randomized trial includes internationally recognized and leading experts in PAD, mitochondrial activity, skeletal muscle, functional performance, and randomized trial design and statistical analyses. Members of the investigative team have been working together for up to 20 years on NIH-funded and PCORI funded studies to identify clinical characteristics associated with functional decline in PAD and more recently to identify novel therapies to improve functional performance and prevent functional decline in people with PAD.

The investigative team is internationally recognized for its experience and expertise successfully completing randomized clinical trials. Since 2009, the investigative team, led by Dr. McDermott, has completed enrollment for six randomized trials of participants with PAD that were funded by NIH or PCORI.

The investigative team has substantial experience with the recruitment methods described above. Between January 1, 2015 and July 31, 2017, we randomized 466 eligible participants with PAD into our randomized clinical trials at Northwestern: an average of 14.6 participants per month.

Health interviewers will be trained by a senior coordinator and certified by Dr. McDermott in each data collection element, using a detailed checklist developed for the NICE Trial. Health interviewers are re-certified approximately every six months. When deficiencies are identified, interviewers undergo additional training and re-assessment.

Baseline and follow-up data collection will take place at Northwestern Memorial Hospital in the Division of General Internal Medicine space located on the 11th and 18th floors of the Galter Pavilion (675 N. St. Clair) or at 750 N. Lake Shore. Treadmill stress testing will be performed by Northwestern Medicine on the 8th floor of the Feinberg Pavilion (251 E. Huron).

RESULTS. Number of participants who began the study run-in and did not meet adherence criteria required for randomization
N=104 participants started run-in, among those, N=8 (7.7%) did not successfully complete run-in.

Supplementary Table 1. List of number (percent) of run-in pills taken by participants who did not successfully complete run-in

Obs	ID	NR/Placebo pills taken	NR adherence	Resveratrol/Placebo pills taken	Resveratrol adherence
**1**	N1310	28	50.0%	7	50.0%
**2**	N1568	Pill counts could not be retrieved for this participant.			
**3**	N2072	2	3.6%	1	7.1%
**4**	N2402	20	35.7%	10	71.4%
**5**	N2419	36	64.3%	9	64.3%
**6**	N2930	20	35.7%	5	35.7%
**7**	N2995	38	67.9%	9	64.3%
**8**	N3113	24	42.9%	14	100.0%

Supplementary Table 2. Effects of nicotinamide riboside and nicotinamide riboside + resveratrol on changes in exploratory outcomes for the NICE randomized clinical trial

	Nicotinamide Riboside (NR) (N=28)			Nicotinamide Riboside (NR) + Resveratrol (R) (N=33)			Placebo (N=28)				
	Baseline, Mean (SD)	6-month follow-up LSMeans	6-month within-group change, LSMeans (SE)	Baseline, Mean (SD)	6-month follow-up LSMeans	6-month within-group change, LSMeans (SE)	Baseline, Mean (SD)	6-month follow-up LSMeans	6-month within-group change, LSMeans (SE)	NR vs. placebo, (one-sided 90% CI)	NR + R vs. placebo, (one-sided 90% CI)
**Walking Impairment Questionnaire speed score**	47.01 (27.16)	47.23	0.22 (3.43)	41.40 (25.52)	40.58	-0.82 (3.08)	44.18 (20.44)	47.11	2.93 (3.34)	−2.71 (−8.97, +∞) P=0.71	-3.75 (−9.63, +∞) P=0.79
**Walking Impairment Questionnaire stair climbing score**	56.10 (27.18)	58.19	2.09 (3.55)	57.70 (31.32)	55.06	-2.64 (3.18)	54.02 (29.65)	51.87	-2.15 (3.46)	4.24 (−2.25, +∞) P=0.20	-0.48 (−6.57, +∞) P=0.54
**Short-Form-36 physical functioning score**	54.82 (24.81)	51.42	-3.40 (3.23)	54.24 (22.99)	54.89	0.65 (2.89)	57.32 (20.30)	61.32	4.00 (3.15)	−7.40 (−13.31, +∞) P=0.94	-3.35 (−8.89, +∞) P=0.78

The Walking Impairment Questionnaire and Short-Form-36 questionnaires are scored on a 0-100 scale (100-best). Statistical analyses were performed using analysis of covariance (ANCOVA), adjusting for baseline values for each outcome, age, sex, and race. The *a priori* statistical analysis plan defined a one-sided P value of < 0.10 to define statistical significance.

Supplementary Table 3. Effects of nicotinamide riboside and nicotinamide riboside + resveratrol combined on changes in exploratory outcomes, compared to placebo, for the NICE randomized clinical trial

	NR alone and NR + resveratrol groups combined (N=61)			Placebo (N=28)			Comparison of change between the combined NR groups and placebo
	Baseline, Mean (Standard Deviation)	Follow-up, LSMeans	Within group change, LSMeans (Standard Error)	Baseline, Mean (SD)	Follow-up, LSMeans	Within group change, LSMeans (Standard Error)	P value
Walking impairment questionnaire speed score	43.98 (26.22)	43.68	-0.30 (2.28)	44.18 (20.44)	46.59	2.41 (4.10)	-2.71 (-8.97, +∞) P=0.71
Walking impairment questionnaire stair climbing score	56.97 (29.26)	56.70	-0.27 (2.36)	54.02 (29.65)	49.51	-4.51 (4.25)	4.24 (-2.25, +∞) P=0.20
Short-form 36 physical functioning score	54.51 (23.64)	53.14	-1.37 (2.15)	57.32 (20.30)	63.34	6.02 (3.87)	-7.40 (-13.31, +∞) P=0.94

The Walking Impairment Questionnaire and Short-Form-36 questionnaires are scored on a 0-100 scale (100-best). Statistical analyses were performed using analysis of covariance (ANCOVA), adjusting for baseline values for each outcome, age, sex, and race. The *a priori* statistical analysis plan defined a one-sided P value of < 0.10 to define statistical significance.

Supplementary Table 4. Effects of nicotinamide riboside + resveratrol, compared to nicotinamide riboside, on exploratory outcomes in the NICE Trial.

	NR + resveratrol group (N=33)			Nicotinamide riboside (N=28)			Comparison of change between the NR + resveratrol vs. NR
	Baseline, Mean (Standard Deviation)	Follow-up, LSMeans	Within group change, LSMeans (Standard Error)	Baseline, Mean (Standard Deviation)	Follow-up, LSMeans	Within group change, LSMeans (Standard Error)	P value
Walking impairment questionnaire speed score	41.40 (25.52)	40.58	-0.82 (3.08)	47.01 (27.16)	47.23	0.22 (3.43)	−1.04 (−7.07, +∞) P=0.59
Walking impairment questionnaire stair climbing score	57.70 (31.32)	55.06	-2.64 (3.18)	56.10 (27.18)	58.19	2.09 (3.55)	−4.72 (−10.94, +∞) P=0.84
Short-form 36 physical functioning score	54.24 (22.99)	54.89	0.65 (2.89)	54.82 (24.81)	51.42	-3.40 (3.23)	4.05 (−1.6[illegible], +∞) P=0.1[illegible]

The Walking Impairment Questionnaire and Short-Form-36 questionnaires are scored on a 0-100 scale (100-best). Statistical analyses were performed using analysis of covariance (ANCOVA), adjusting for baseline values for each outcome, age, sex, and race. The *a priori* statistical analysis plan defined a one-sided P value of < 0.10 to define statistical significance.

Supplementary Table 5. Rates of adverse events by study group

Adverse event	Trial Assignment		
	**NR+Resveratrol (# of participants=33)**	**NR alone (# of participants=28)**	**Placebo (# of participants=29)**
Nausea or vomiting, n (%)	12 (36.4%)	4 (14.3%)	7 (24.1%)
Diarrhea, n (%)	18 (54.6%)	11 (39.3%)	8 (27.6%)
Abdominal discomfort, n (%)	13 (39.4%)	11 (39.3%)	13 (44.8%)
Flushing, n (%)	9 (27.3%)	9 (32.1%)	10 (34.5%)
Participants who reported at least one adverse events, n (%)	24 (72.7%)	18 (64.3%)	21 (72.4%)

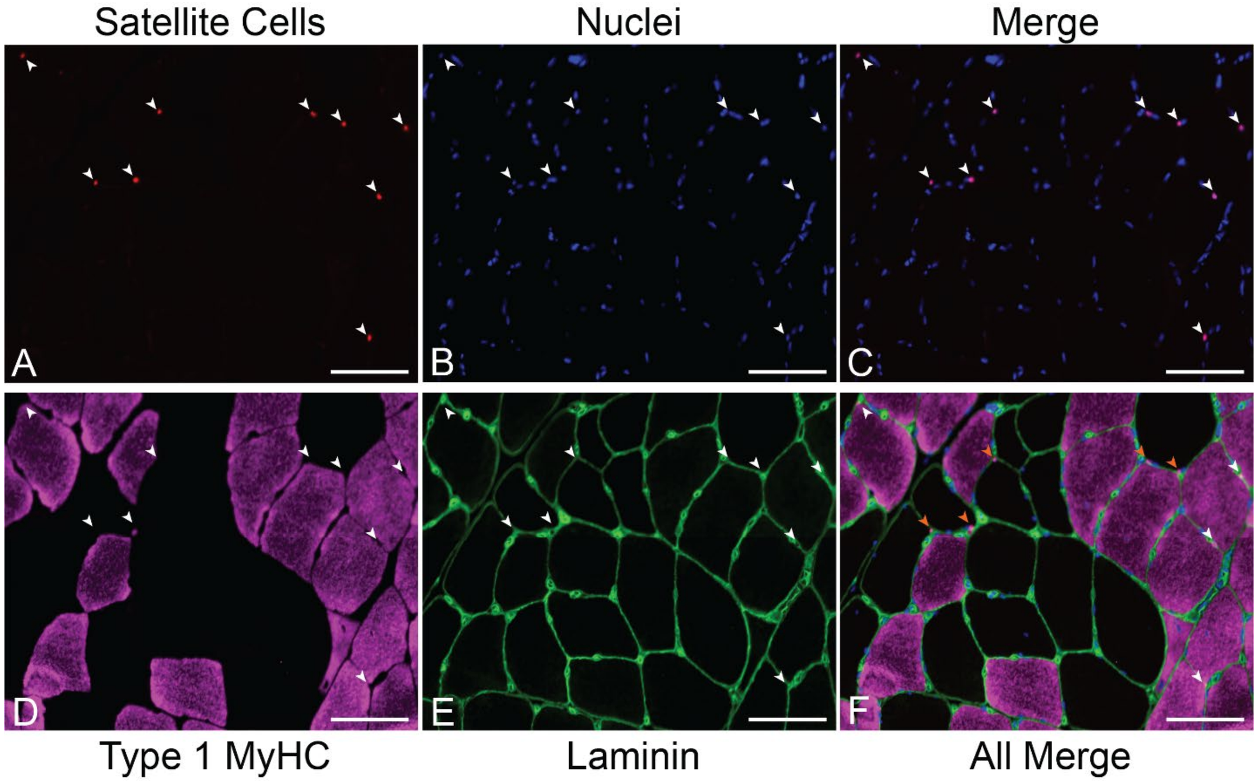

Supplementary Figure 1.
A-C. Representative images showing satellite cells (white arrows) identified by expression of Pax7 (red, A) and DAPI-labeled DNA (blue, B); merge image (C) showing satellite cells are Pax7+/DAPI+. D. Image of muscle fibers expressing Type I myosin heavy chain (MyHC, pink). E. Fiber borders demarcated by laminin (green). F. Merge of all labels showing satellite cells associated with both Type 1 fibers (white arrows) and Type 2 fibers (orange arrows). Regions of interest acquired at 200x, scale bar = 100 µm.

REFERENCES

1.McDermott MM, Greenland P, Liu K, Guralnik JM, Criqui MH, Dolan NC, Chan C, Celic L, Pearce WH, Schneider JR, Sharma L, Clark E, Gibson D, Martin GJ. Leg symptoms in peripheral arterial disease: Associated clinical characteristics and functional impairment. *JAMA.* 2001;286:1599-1606.

2. McDermott MM, Greenland P, Liu K, Guralnik JM, Celic L, Criqui MH, Chan C, Martin GJ, Schneider J, Pearce WH, Taylor LM, Clark E. The ankle brachial index is associated with leg function and physical activity: The Walking and Leg Circulation Study. *Ann Intern Med.* 2002;136:873-83.

3. McDermott MM, Liu K, Greenland P, Guralnik JM, Criqui MH, Chan C, Pearce WH, Schneider JR, Ferrucci L, Celic L, Taylor LM, Vonesh E, Martin GJ, Clark E. Functional decline in peripheral arterial disease: Associations with the ankle brachial index and leg symptoms. *JAMA.* 2004;292:453-61.

4. McDermott MM, Guralnik JM, Tian L, Liu K, Ferrucci L, Liao Y, Sharma L, Criqui MH. Associations of borderline and low normal ankle-brachial index values with functional decline at 5-year follow-up: The WALCS (Walking and Leg Circulation Study). *J Am Coll Cardiol.* 2009;53:1056-62.

5. McDermott MM, Guralnik JM, Tian L, Ferrucci L, Liu K, Liao Y, Criqui MH. Baseline functional performance predicts the rate of mobility loss in persons with peripheral arterial disease. *J Am Coll Cardiol*. 2007;50:974-82.

6. McDermott MM, Fried L, Simonsick E, Ling S, Guralnik JM. Asymptomatic peripheral arterial disease is independently associated with impaired lower extremity functioning: The women's health and aging study. *Circulation.* 2000;101:1007-12.

7. Weiss DJ, Casale GP, Koutakis P, Nella AA, Swanson SA, Zhu Z, Miserlis D, Johanning JM, Pipinos II. Oxidative damage and myofiber degeneration in the gastrocnemius of patients with peripheral arterial disease. *J Transl Med.* 2013;11:230-9.

8. Gürke L, Marx A, Sutter PM, Stierli P, Harder F, Heberer M. Function of fast- and slow-twitch rat skeletal muscle following ischemia and reperfusion at different intramuscular temperatures. *Eur Surg Res.* 2000;32:135-41.

9. Gillani S, Cao J, Suzuki T, Hak DJ. The effect of ischemia reperfusion injury on skeletal muscle. *Injury.* 2012;43:670-5.

10. Pipinos II, Sharov VG, Shepard AD, Anagnostopoulos PV, Katsamouris A, Todor A, Filis KA, Sabbah HN. Abnormal mitochondrial respiration in skeletal muscle in patients with peripheral arterial disease. *J Vasc Surg.* 2003;38:827-32.

11. Pipinos II, Judge AR, Selsby JT, Zhu Z, Swanson SA, Nella AA, Dodd SL. The myopathy of peripheral arterial occlusive disease: Part 2. Oxidative stress, neuropathy, and shift in muscle fiber type. *Vasc Endovascular Surg.* 2008;42:101-12.

12. Pipinos II, Swanson SA, Zhu Z, Nella AA, Weiss DJ, Gutti TL, McComb RD, Baxter BT, Lynch TG, Casale GP. Chronically ischemic mouse skeletal muscle exhibits myopathy in association with mitochondrial dysfunction and oxidative damage. *Am J Physiol Regul Integr Comp Physiol.* 2008;295:R290-6.

13. Rontoyanni V, Lopez ON, Fankhauser GT, Cheema ZF, Rasmussen BB, Porter C. Mitochondrial bioenergetics in the metabolic myopathy accompanying peripheral artery disease. *Front Physiol.* 2017;8:1-12.

14. Ueta CB, Gomes KA, Riberio MA, Mochly-Rosen D, Ferreira JCB. Disruption of mitochondrial quality control in peripheral artery disease: New therapeutic opportunities. *Pharmacol Res.* 2017;115:96-106.

15. Hou XY, Green S, Askew CD, Barker G, Green A, Walker PJ. Skeletal muscle mitochondrial ATP production rate and walking performance in peripheral arterial disease. *Clin Physiol Funct Imaging.* 2002;22:226-32.

16. McDermott MM, Ferrucci L, Guralnik J, Tian L, Liu K, Hoff F, Liao Y, Criqui MH. Pathophysiological changes in calf muscle predict mobility loss at 2-year follow-up in men and women with peripheral arterial disease. *Circulation.* 2009;120:1048-55.

17. Anderson JD, Epstein FH, Meyer CH, Hagspiel KD, Wang H, Berr SS, Harthun NL, Weltman A, Dimaria JM, West AM, Kramer CM. Multifactorial determinants of functional capacity in peripheral arterial disease: uncoupling of calf muscle perfusion and metabolism. *J Am Coll Cardiol.* 2009;54:628-35.

18. McDermott MM, Hoff F, Ferrucci L, Pearce WH, Guralnik JM, Tian L, Liu K, Schneider JR, Sharma L, Tan J, Criqui MH. Lower extremity ischemia, calf skeletal muscle characteristics, and functional impairment in peripheral arterial disease. *J Am Geriatr Soc.* 2007;55:400-6.

19. Trammell SAJ, Schmidt MS, Weidemann BJ, Redpath P, Jaksch F, Dellinger RW, Li Z, Abel, ED, Migaud ME, Brenner C. Nicotinamide riboside is uniquely and orally bioavailable in mice and humans. *Nat Commun*. 2016;7:12948.

20. Massudi H, Grant R, Braidy N, Guest J, Farnsworth B, Guillemin GJ. Age-associated changes in oxidative stress and NAD+ metabolism in human tissue. *PloS one*. 2012;7:e42357.

21. Khan NA, Auranen M, Paetau I, Pirinen E, Euro L, Forsström S, Pasila L, Velagapudi V, Carroll CJ, Auwerx J, Suomalainen A. Effective treatment of mitochondrial myopathy by nicotinamide riboside, a vitamin B3. *EMBO Mol Med*. 2014;6:721-31.

22. Bonkowski MS, Sinclair DA. Slowing ageing by design: the rise of NAD+ and sirtuin-activating compounds. *Nat Rev Mol Cell Biol*. 2016;17: 679-90.

23. Kim JS, Chung-Su Y, Park DR. NAMPT regulates mitochondria biogenesis via NAD metabolism and calcium binding proteins during muscle contraction. *J Exerc Nutrition Biochem*. 2014;18:259-66.

24. Imai S, Yoshino J. The importance of NAMPT/NAD/SIRT1 in the systemic regulation of metabolism and ageing. *Diabetes Obes Metab*. 2013;15 Suppl 3:26-33.

25. Li L, Pan R, LI R, Niemann B, Aurich AC, Chen Y, Rohrbach S. Mitochondrial biogenesis and peroxisome proliferator–Activated Receptor-γ Coactivator-1α (PGC-1α) Deacetylation by Physical Activity. *Diabetes*. 2011;60:157-67.

26. Ryall JG, Dell'Orso S, Derfoul A, Juan A, Zare H, Feng X, Clermont D, Koulnis M, Gutierrez-Cruz G, Fulco M, Sartorelli V. The NAD+-dependent SIRT1 deacetylase translates a metabolic switch into regulatory epigenetics in skeletal muscle stem cells. *Cell Stem Cell.* 2015;16:171-83.

27. Zhang QJ, Wang Z, Chen HZ, Zhou S, Zheng W, Lui G, Wei YS, Cai H, Liu DP, Liang CC. Endothelium-specific overexpression of class III deacetylase SIRT1 decreases atherosclerosis in apolipoprotein E-deficient mice. *Cardiovasc Res*. 2008;80:191-9.

28. Stein S, Schafer N, Breitenstein A, Besler C, Winnik S, Lohmann C, Heinrich K, Brokopp CE, Handschin C, Landmesser U, Tanner FC, Luscher TF, Matter CM. SIRT1 reduces endothelial activation without affecting vascular function in ApoE-/- mice. *Aging (Albany NY).* 2010;2:353-60.

29. Baur JA, Pearson KJ, Price NL, Jamieson HA, Lerin C, Kalra A, Prabhu VV, Allard JS, Lopez-Lluch G, Lewis K, Pistell PJ, Poosala S, Becker KG, Boss O, Gwinn D, et al. Resveratrol improves health and survival of mice on a high-calorie diet. *Nature*. 2006;444:337-342.

30. Lagouge M, Argmann C, Gerhart-Hines Z, Meziane H, Lerin C, Daussin F, Messadeq N, Milne J, Lambert P, Elliott P, Geny B, Laakso M, Puigserver P, Auwerx J. Resveratrol improves mitochondrial function and protects against metabolic disease by activating SIRT1 and PGC-1α. *Cell*. 2006;127:1109-22.

31. Cantó C, Houtkooper RH, Pirinen E, Youn DY, Oosterveer MH, Cen Y, Fernandez-Marcos PJ, Yamamoto H, Andreux PA, Cettour-Rose P, Gademann K, Rinsch C, Schoonjans K, Sauve AA, Auwerx J. The NAD+ precursor nicotinamide riboside enhances oxidative metabolism and protects against high-fat diet-induced obesity. *Cell Metab*. 2012;15:838-47.

32. Zhang H, Ryu D, Wu Y, Gariani K, Wang X, Luan P, D'Amico D, Ropelle ER, Lutolf MP, Aebersold R, Schoonjans K, Menzies KJ, Auwerx J. NAD+ repletion improves mitochondrial and stem cell function and enhances life span in mice. *Science*. 2016;352:1436-43.

33. Gomes AP, Price NL, Ling AJ, Moslehi JJ, Montgomery MK, Rajman L, White JP, Teodoro JS, Wrann CD, Hubbard BP, Mercken EM, Palmeira CM, de Cabo R, Rolo AP, Turner N et al. Declining NAD(+) induces a pseudohypoxic state disrupting nuclear-mitochondrialcommunication during aging. *Cell*. 2013;155:1624-38.

34. Yang Y, Sauve AA. NAD+ metabolism: Bioenergetics, signaling and manipulation for therapy. *Biochim Biophys Acta*. 2016;1864:1787-1800.

35. Yoshino J, Mills KF, Yoon, MJ, Imai SI. Nicotinamide mononucleotide, a key NAD+ intermediate, treats the pathophysiology of diet-and age-induced diabetes in mice. *Cell Metab*. 2011;14:528-536.

36. Sin TK, Yung BY, Siu PM. Modulation of SIRT1-Foxo1 signaling axis by resveratrol: implications in skeletal muscle aging and insulin resistance. *Cell Physiol Biochem*. 2015;35:541-52.

37. Price, NL, Gomes AP, Ling AJ, Duarte FV, Martin-Montalvo A, North BJ, Agarwal B, Ye L, Ramadori G, Teodoro, JS, Hubbard, BP, Varela, AT, Davis JG, Varamini B, Hafner A. et al. SIRT1 is required for AMPK activation and the beneficial effects of resveratrol on mitochondrial function. *Cell Metab.* 2012;15: 675-90.

38. Zhang Y, Tian F, Xiao Q, Hu Y, Li J, Jiang F, Liu Y. Exploiting the Role of Resveratrol in Rat Mitochondrial Permeability Transition. *J Membr Biol*. 2013;246:365-73.

39. Chen S, Fan Q, Li A, Liao D, Ge J, Laties AM, Zhang X. Dynamic mobilization of PGC-1α mediates mitochondrial biogenesis for the protection of RGC-5 cells by resveratrol during serum deprivation. *Apoptosis.* 2013;18:786-99.

40. Menzies KJ, Singh K, Saleem A, Hood DA. Sirtuin 1-mediated effects of exercise and resveratrol on mitochondrial biogenesis. *J Biol Chem.* 2013; 288:6968-79.

41. Lin Y, Chen F, Zhang J, Wang T, Wei X, Wu J, Feng Y, Dai Z, Wu Q. Neuroprotective effect of resveratrol on ischemia/reperfusion injury in rats through TRPC6/CREB pathways. *J Mol Neurosci.* 2013;50:504-13.

42. Shen M, Wu RX, Zhao L, Li J, Guo HT, Fan R, Cui Y, Wang YM, Yue SQ, Pei JM. Resveratrol attenuates ischemia/reperfusion injury in neonatal cardiomyocytes and its underlying mechanism. *PLoS One.* 2012;7:e51223.

43. Howitz KT, Bitterman KJ, Cohen HY, Lamming DW, Lavu S, Wood JG, Zipkin RE, Chung P, Kisielewski A, Zhang L, Scherer B, Sinclair DA. Small molecule activators of sirtuins extend saccharomyces cerevisiae lifespan. *Nature*. 2003;425:191-6.

44. McDermott MM, Leeuwenburgh C, Guralnik JM, Tian L, Sufit R, Zhao L, Criqui MH, Kibbe MR, Stein JH, Lloyd-Jones D, Anton SD, Polonsky TS, Gao Y, de Cabo R, Ferrucci L. Effect of resveratrol on walking performance in older people with peripheral artery disease. The RESTORE randomized clinical trial. *JAMA Cardiol*. 2017;2:902-7.

45. Go AS, Mozaffarian D, Roger VL, Benjamin EJ, Berry JD, Borden WB, Bravata DM, Dai S, Ford ES, Fox CS, Franco S, Fullerton HJ, Gillespie C, Hailpern SM, Heit JA, et al. Heart disease and stroke statistics—2013 update: a report from the American Heart Association. *Circulation.* 2013;127:e841.

46. Fowkes FG, Rudan D, Rudan I, Aboyans V, Denenberg JO, McDermott MM, Norman PE, Sampson UK, Williams LJ, Mensah GA, Criqui MH. Comparison of global estimates of prevalence and risk factors for peripheral artery disease in 2000 and 2010: A systematic review and analysis. *Lancet.* 2013;382:1329-40.

47. Fried LP, Guralnik JM. Disability in older adults: Evidence regarding significance, etiology, and risk. *J Am Geriatr Soc.* 1997;45:92-100.

48. Guralnik JM, Simonsick EM, Ferrucci L, Glynn RJ, Berkman LF, Blazer DG, Scherr PA, Wallace RB. A short physical performance battery assessing lower extremity function: Association with self-reported disability and prediction of mortality and nursing home admission. *J Gerontol.* 1994;49:M85-94.

49. Guralnik JM, Ferrucci L, Simonsick EM, Salive ME, Wallace RB. Lower-extremity function in persons over the age of 70 years as a predictor of subsequent disability. *N Engl J Med.* 1995;332:556-61.

50. Murray CJ, Atkinson C, Bhalla K, Birbeck G, Burstein R, Chou D, Dellavalle R, Danaei G, Ezzati M, Fahimi A, Flaxman D, Foreman, Gabriel S, Gakidou E, Kassebaum N, et al. The state of US health, 1990-2010: Burden of diseases, injuries, and risk factors. *JAMA.* 2013;310:591-608.

51. Gerhard-Herman MD, Gornik HL, Barrett C, Barshes NR, Corriere MA, Drachman DE, Fleisher LA, Fowkes FG, Hamburg NM, Kinlay S, Lookstein R, Misra S, Mureebe L, Olin JW, Patel RA, et al. 2016 AHA/ACC Guideline on the Management of Patients With Lower Extremity Peripheral Artery Disease: A Report of the American College of Cardiology/American Heart Association Task Force on Clinical Practice Guidelines. *J Am Coll Cardiol*. 2017;69:e71-e126.

52. Stevens JW, Simpson E, Harnan S, Squires H, Meng Y, Thomas S, Michaels J, Stansby G. Systematic review of the efficacy of cilostazol, naftidrofuryl oxalate and pentoxifylline for the treatment of intermittent claudication. *Br J Surg.* 2012;99:1630-8.

53. Dawson DL, Cutler BS, Meissner MH, Strandness DE Jr. Cilostazol has beneficial effects in treatment of intermittent claudication: results from a multicenter, randomized, prospective, double-blind trial. *Circulation.* 1998;98:678-86.

54. Money SR, Herd JA, Isaacsohn JL, Davidson M, Cutler B, Heckman J, Forbes WP. Effect of cilostazol on walking distances in patients with intermittent claudication caused by peripheral vascular disease. *J Vasc Surg.* 1998;27:267-74.

55. Beebe HG, Dawson DL, Cutler BS, Herd JA, Strandness DE Jr, Bortey EB, Forbes WP. A new pharmacological treatment for intermittent claudication: Results of a randomized, multicenter trial. *Arch Intern Med.* 1999;159:2041-50.

56. Dormandy JA, Rutherford RB. Management of Peripheral Arterial Disease (PAD). TASC Working Group. TransAtlantic Inter-Society Consensus (TASC). *J Vasc Surg*. 2000;31(1 Pt 2): S1-S296.

57. Jones DW, Schanzer A, Zhao Y, MacKenzie TA, Nolan BW, Conte MS, Goodney PP, Vascular Study Group of New England. Growing impact of restenosis on the surgical treatment of peripheral arterial disease. *J Am Heart Assoc.* 2013;2:e000345.

58. McDermott MM. Exercise training for intermittent claudication. J Vasc Surg 2017; doi: 10.1016/j.jvs.2017.05.111.

59. Harwood AE, Smith GE, Cayton T, Broadbent E, Chetter IC. A systematic review of the uptake and adherence rates to supervised exercise programs in patients with intermittent claudication. *Ann Vasc Surg*. 2016;34:280-9.

60. Ryan TE, Schmidt CA, Green TD, Spangenburg EE, Neufer PD, McClung JM. Targeted expression of catalase to mitochondria protects against ischemic myopathy in high fat fed mice. *Diabetes*. 2016;65:2553-68.

61. Ryan TE, Schmidt CA, Alleman RJ, Tsang AM, Green TD, Neufer PD, Brown DA, McClung JM. Mitochondrial therapy improves limb perfusion and myopathy following hindlimb ischemia. *J Mol Cell Cardiol*. 2017;97:191-6.

62. Nisoli E, Tonello C, Cardile A, Cozzi V, Bracale R, Tedesco L, Falcone S, Valerio A, Cantoni O, Clementi E, Moncada S, Carruba MO. Calorie restriction promotes mitochondrial biogenesis by inducing the expression of eNOS. *Science*. 2005;310:314-17.

63. Winnik S, Auwerx J, Sinclair D, Matter CM. Protective effects of sirtuins in cardiovascular diseases: from bench to bedside. *Eur Heart J*. 2015;36:3404-12.

64. Rowe GC, Jiang A, Arany Z, Kelly DP. PGC-1 coactivators in cardiac development and disease. *Circ Res*. 2010;10:825-838.

65. Martens CR, Denman BA, Mazzo MR, Armstrong M, Reisdorph N, McQueen MB, Chonchol M, Seals DR. Nicotinamide riboside safely and effectively raises NAD+ and improves cardiovascular function in middle aged/older adults. Science Research Conferences. Federation of American Societies for Experimental Biology. Abstract, July 9, 2017.

66. Timmers S, Konings E, Bilet L, Houtkooper RH, van de Weijer T, Goossens GH, Hoeks J, van der Krieken S, Ryu D, Kersten S, Moonen-Kornips E, Hesselink MK, Kunz I, Schrauwen-Hinderling VB, Blaak EE, et al. Calorie restriction-like effects of 30 days of resveratrol supplementation on energy metabolism and metabolic profile in obese humans. *Cell Metab*. 2011;14:612-22.

67. Mattison JA, Wang M, Bernier M, Zhang J, Park SS, Maudsley S, An SS, Santhanam L, Martin B, Faulkner S, Morrell C, Baur JA, Peshkin L, Sosnowska D, Csiszar A, et al. Resveratrol prevents high fat/sucrose diet-induced central arterial wall inflammation and stiffening in nonhuman primates. *Cell Metab*. 2014;20:183-90.

68. Juhasz B, Mukherjee S, Das DK. Hormetic response of resveratrol against cardioprotection. *Exp Clin Cardiol.* 2010;15:134-138.

69. Baur JA, Pearson KJ, Price NL, Jamieson HA, Lerin C, Kalra A, Prabhu VV, Allard JS, Lopez-Llurich G, Lewis K, Pistell PJ, Poosala S, Becker KG, Boss O, Gwinn D, et al. Resveratrol improves health and survival of mice on a high-calorie diet. *Nature.* 2006;444:337-42.

70. Price NL, Gomes AP, Ling AJ, Duarte FV, Martin-Montalvo A, North BJ, Agarwal B, Ye L, Ramadori G, Teodoro JS, Hubbard BP, Varela AT, Davis JG, Varamini B, Hafner A, et al. SIRT1 is required for AMPK activation and the beneficial effects of resveratrol on mitochondrial function. *Cell Metab*. 2012;15:675-90.

71. Gu J, Wang CQ, Fan HH, Ding HY, Xie XL, Xu YM, Wang BY, Huang DJ. Effects of resveratrol on endothelial progenitor cells and their contributions to reendothelialization in intima-injured rats. *J Cardiovasc Pharmacol.* 2006;47:711-21.

72. Gurusamy N, Lekli I, Mukherjee S, Ray D, Ahsan MK, Gherghiceanu M, Popescu LM, Das DK. Cardioprotection by resveratrol: A novel mechanism via autophagy involving the mTORC2 pathway. *Cardiovascular Res.* 2010;86:103-12.

73. Kumar V, Pandey A, Jahan S, Shukla RK, Kumar D, Srivastava A, Singh S, Rajpurohit CS, Yadav S, Khanna VK, Pant AB. Differential responses of Trans-Resveratrol on proliferation of neural progenitor cells and aged rat hippocampal neurogenesis. *Sci Rep.* 2016;6:28142.

74. McDermott MM, Liu K, Guralnik JM, Criqui MH, Spring B, Tian L, Domanchuk K, Ferrucci L, Lloyd-Jones D, Kibbe M, Tao H, Zhao L, Liao Y, Rejeski J. Home-based walking exercise intervention in peripheral artery disease: a randomized clinical trial. *JAMA*. 2013;310:57-65.

75. White S, McDermott MM, Sufit RL, Kosmac K, Bugg AW, Gonzalez-Freire M, Ferrucci L, Tian L, Zhao L, Gao Y, Kibbe MR, Criqui MH, Leeuwenburgh C, Peterson CA. Walking performance is positively correlated to calf muscle fiber size in peripheral artery disease subjects, but fiber show abberant mitophagy: an observational study. *J Transl Med*. 2016;14:284-299.

76. Vang O, Ahmad N, Baile CA, Baur JA, Brown K, Csiszar A, Das DK, Delmas D, Gottfried C, Lin HY, Ma QY, Mukhopadhyay P, Nalini N, Pezzuto JM, et al. What is new for an old molecule? Systematic review and recommendations on the use of resveratrol. *PloS One*. 2011;6:e19881.

77. Perera S, Mody SH, Woodman RC, Studenski SA. Meaningful change and responsiveness in common physical performance measures in older adults. *J Am Geriatr Soc.* 2006;54:743-9.

78. Puhan MA, Mador MJ, Held U, Goldstein R, Guyatt GH, Schünemann HJ. Interpretation of treatment changes in 6-minute walk distance in patients with COPD. *Eur Respir J.* 2008;32:637–43.

79. Holland AE, Hill CJ, Conron M, Munro P, McDonald CF. Small changes in six-minute walk distance are important in diffuse parenchymal lung disease. *Respir Med*. 2009;103:1430-5.

80. Schrover R, Evans K, Giugliani R, Noble I, Bhattacharya K. Minimal clinically important difference for the 6-min walk test: literature review and application to Morquio A syndrome. *Orphanet J Rare Dis*. 2017;12:78.

81. Peek CB, Affinati AH, Ramsey KM, Kuo HY, Yu W, Sena LA, Iikayeva O, Marcheva B, Kobayashi Y, Omura C, Levine DC, Bacsik DJ, Gius D, Newgard CB, Goetzman E, Chandel NS, Denu JM, Mrksich M, Bass J. Circadian clock NAD+ cycle drives mitochondrial oxidative metabolism in mice. Science 2013 342(6158):1243417.

82. Fry, CS., B. Noehren, J. Mula, M.F. Ubele, P.M. Westgate, P.A. Kern, C.A. Peterson. Fiber type-specific satellite cell response to aerobic training in sedentary adults. *J. Physiol*. 2014; 592:2625-36.

83. Sagar SP, Brown PM, Zelt DT, Pickett WL, and Tranmer JE. Further Clinical Validation of the Walking Impairment Questionnaire for Classification of Walking Performance in Patients with Peripheral Artery Disease. *Int J Vasc Med*. 2012;doi:10 1155/2012/190641.
